# Design, synthesis and bioactive properties of a class of macrocycles with tunable functional groups and ring size

**DOI:** 10.1038/s41598-022-08775-z

**Published:** 2022-03-21

**Authors:** Liya Thurakkal, Pandurangan Nanjan, Mintu Porel

**Affiliations:** Department of Chemistry, Indian Institute of Technology Palakkad; Environmental Sciences and Sustainable Engineering Center, Indian Institute of Technology Palakkad, Palakkad, Kerala 678557 India

**Keywords:** Chemistry, Supramolecular chemistry, Chemical synthesis

## Abstract

The design and synthesis of a versatile class of macrocycles with tunable functional groups and ring size are unfolded. Herein, a synthetic strategy is reported to furnish a new class of macrocycles in multi-gram scale in a two-step reaction. The total time taken for synthesizing a macrocycle is 1.5 h. Dithiocarbamates, an important functional group in biomedical and material sciences, is strategically incorporated in the macrocyclic backbone without metal for the first time. It is noteworthy that when state-of-the-art macrocycle synthesis is in millimolar concentration, this work employs the reaction in molar concentration (0.2–0.4 M). As proof-of-principle, a library of macrocycles was synthesized, varying the functional groups and ring size. The physicochemical properties of macrocycles revealed their druggable nature and are affirmed by protein (serum albumin) interaction study theoretically and experimentally. Diverse functional groups and ring sizes of macrocycles brought about twenty-five-fold difference in binding constant with the model protein.

## Introduction

The structural class of cyclic compounds of twelve or more ringed atoms, known as macrocycles are exploited widely in recent years in the area of drug delivery^1,2^, sensors^3^, nanotubes^4^, organo-electronics^5^ and various other fields. The impact of macrocycle in the field of drug discovery is impeccable when some macrocycles exhibit about a thousand times better properties than their structurally similar linear counterparts^6^. For example, macrocyclization resulted in an increase of potency of the compound for about 55,000 times than its linear analogue, which differs only by a methylene group by altering the half-maximal inhibitory concentration (IC_50_) value from the range of few micromolar to nanomolar^6^ in the inhibition of farnesyl transferase against cancer^7^. This is significantly due to the structural preorganization of a macrocycle which achieves the decrease in entropy penalty during binding with the target. Even though many of the natural drugs are macrocycles, there are only a very few synthetic macrocyclic drugs. Along with the deviation from Lipinski’s rule of five^8^, the difficulty in the synthesis of macrocycle limits its potential to be a successful drug candidate in the pharma field.

The foremost hurdle in the macrocycle synthesis is to avoid the possibility of undesirable polymerization. The measures to prevent this phenomenon are tedious and costly in terms of dilution, catalyst and other high-profile strategies. Dilution^9^, phase separation strategy^10^, pseudo high dilution technique^11^ and template-assisted reaction^12^ are the techniques used to synthesize the macrocycle. However, the scalable, efficient and high yield synthetic strategies are still challenging^13^. Herein, we report a two-step synthetic strategy for macrocycle that provides a robust platform for tuning the functional groups and ring size. This strategy also allows for scalability in gram-scale at high concentration (0.2–0.4 M) within 1.5 h. The unique feature of this class of macrocycle is that a biologically important dithiocarbamate group has been strategically incorporated in its backbone.

Dithiocarbamate (DTC) group is in the limelight due to its anticancer^14^, anti-microbial^15,16^, antitripanosomatids^17^, anti-inflammatory^18^ and antileishmanial^19^ properties. A well-known anti-alcohol abuse drug, disulfiram which contains two DTC groups, has been shown to have an anticancer property, and the mechanism involved in it was published in Nature by Skrott et al. In 2017^20^. The incorporation of dithiocarbamate in the macrocycle would enhance the biomedical importance of the compound as it enjoys the flavour of both the macrocycle and dithiocarbamate. In most cases, metal plays a vital role in macrocyclization by coordinate bond formation with free thiol from the dithiocarbamate; thus, all the reported DTC containing macrocycles so far possess a metal in their ring^21–23^. However, so far, there is no reported macrocycle with the dithiocarbamate ester without metals taking a role in the cyclization step. This report puts forth a novel strategy for scalable synthesis of functionality and ring size-tunable macrocycle with dithiocarbamates in the backbone. As the preliminary step towards the journey to be a drug candidate, we studied the interaction of the macrocycles with a model protein serum albumin (used Bovine Serum Albumin (BSA), which has about 76% structural similarity with Human Serum Albumin (HSA)^24^). The promising results guided for designing new candidates and by systematic tuning of the functional group and ring size of the dithiocarbamate—macrocycle showed a substantial change in the binding constant on the experimental studies.

## Results and discussion

### Design and synthesis of macrocycles

In the present work, an attempt was made to design and synthesize a novel class of macrocycles with tunable functional groups and ring size by incorporating dithiocarbamates in the backbone. Functional groups offer modulation in both physical and chemical properties of a compound, and it is noteworthy that mere change in the structure of a compound can be a door to peculiar results in its property. Nonetheless, this kind of modular synthetic strategy for macrocycle is still challenging. We developed the synthetic strategy in such a way that any of the functional groups can be brought into the system depending on the property of interest, and the ring size of the macrocycle can also be tuned. The ring size of a macrocycle is significant when it comes to its application, which involves binding with other entities. It is crucial to control the rigidity or flexibility of the macrocycle as its property is mainly dependent on its conformation and ring size. Among the different methods of cyclization reactions, the bimolecular homodifunctional approach is employed for the synthesis of the macrocycle, where two molecules with two complementary reactive groups are enabled to react to result in a macrocycle.

**Figure 1:**
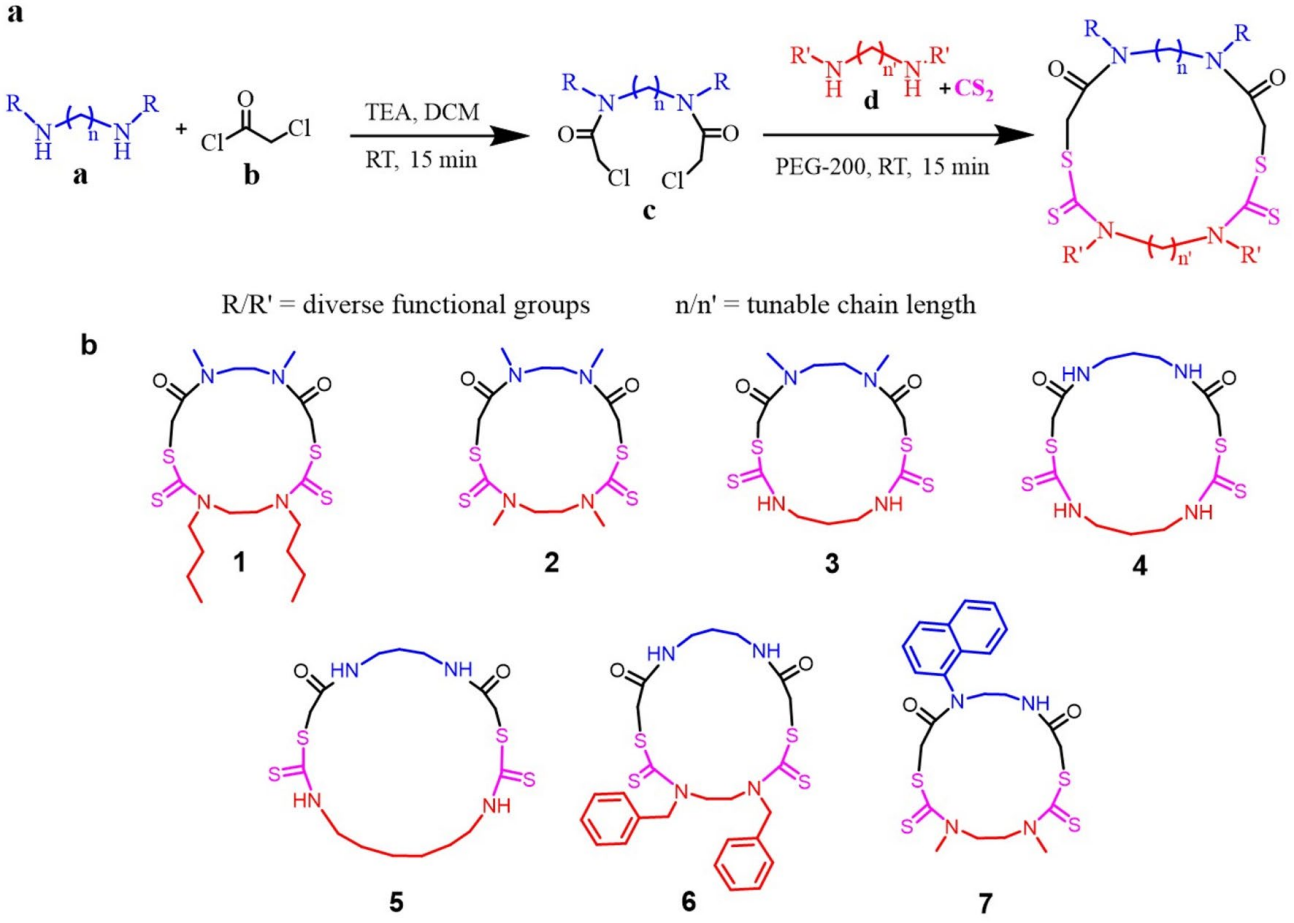
(**a**) Two-step synthetic strategy for macrocyclization. (**b**) Structure of the synthesized macrocycles (*TEA* triethylamine, *DCM* dichloromethane, *RT* room temperature, *PEG* polyethylene glycol).

A two-step reaction involving the inexpensive, readily available starting materials are expended for this strategy. The first step is the reaction of substituted diamine (a, Fig. 1a) with four equivalents of chloroacetyl chloride (b, Fig. 1a) forming a chloro-terminal diamide (c, Fig. 1a). This acid chloride reaction gave quantitative yields within 15 min. The second step is the macrocyclization step which is a three-component reaction between chloro-terminal diamide (c, Fig. 1a), carbon disulfide (CS_2_) and another diamine (d, Fig. 1a) with a desired functional group. In the three-component reaction, an active thiol is formed first by the reaction of diamine (d, Fig. 1a) and CS_2,_ followed by the reaction of thiol with the chloro group of chloro-terminal diamide (c, Fig. 1a). The time taken for the completion of each reaction was 15 min. A kinetics study of the macrocyclization reaction was carried out, and complete conversion of starting material to the product was observed within 15 min (Fig. S2). The solvent used for the macrocyclization was green solvent polyethylene glycol-200 (PEG) which produces significantly fewer environmental hazards, unlike other volatile organic solvents^25^. The optimization of this reaction in various solvents was carried out, and it was observed that in PEG-200, the yield was highest (Table S1). The diamines used for this work were commercially available or synthesized in the lab. This strategy allows many combinations of diamines resulting in macrocycles with different functional groups. For the proof of concept, different functional groups used for this study were hydrogen, methyl, butyl and benzyl and the diamines taken were of varying chain length also. A library of macrocycles was synthesized by combining different functional groups and chain lengths (Fig. 1b). All the macrocycles synthesized for this work resulted in fairly good yield (up to 85%) with different functional groups, properties, and ring sizes.

**Figure 2:**
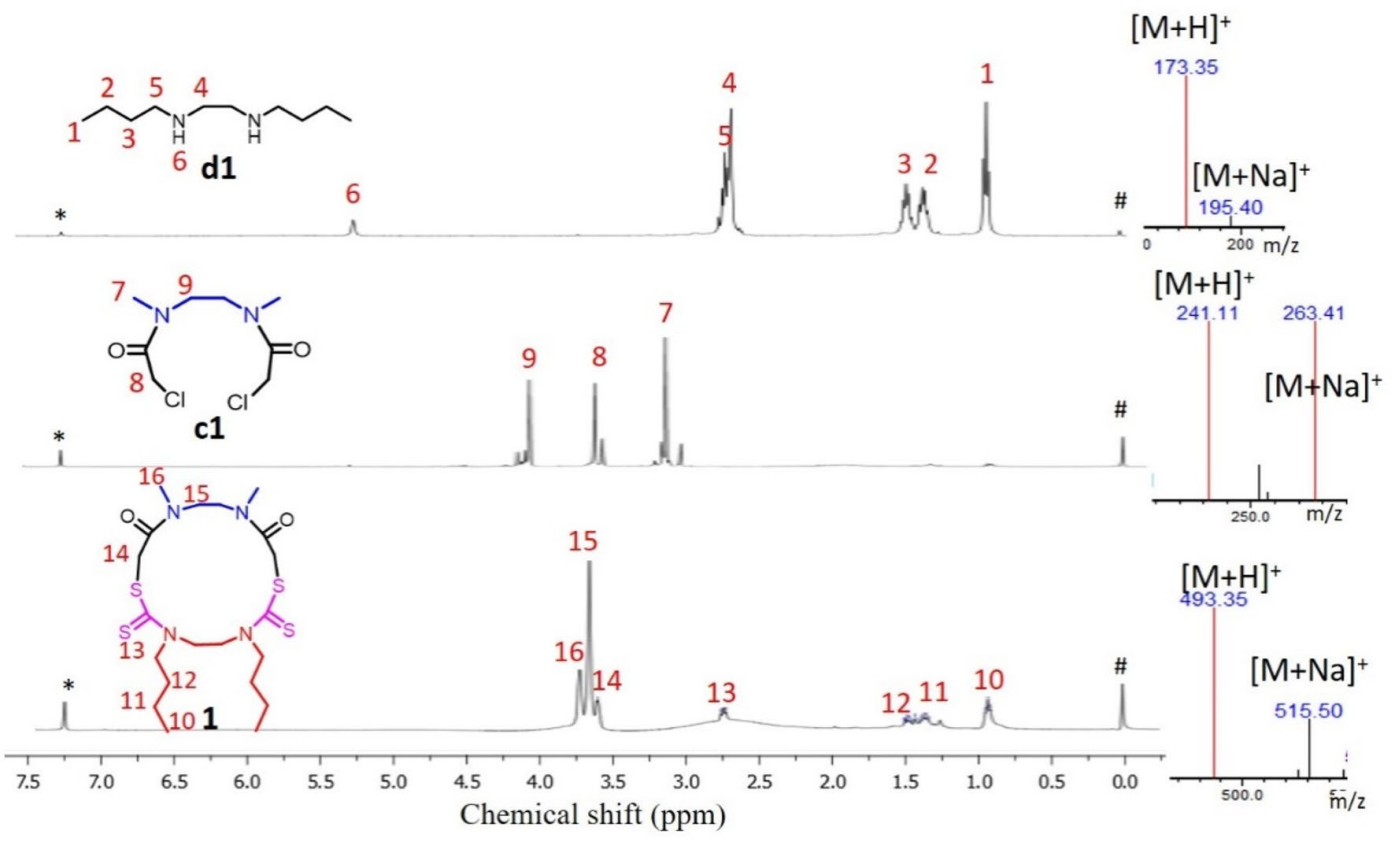
Characterization by ^1^H NMR and LC–MS. *Left* ^1^H NMR of d1, c1 and 1 (“#” and “*” are the residual proton signal of internal standard tetramethylsilane and the solvent CDCl_3_ respectively); *right* Mass spectrum with the peak of [M + H]^+^ and [M + Na]^+^: Calculated [M + H]^+^ for d1: 173.19 Da, observed [M + H]^+^:173.35 Da, [M + Na]^+^: 195.40 Da. Calculated [M + H]^+^ for c1: 241.04 Da, observed [M + H]^+^: 241.11 Da, [M + Na]^+^: 263.41 Da. Calculated [M + H]^+^for 1: 493.17 Da, observed [M + H]^+^: 493.35 Da, [M + Na]^+^: 515.50 Da.

**Figure 3:**
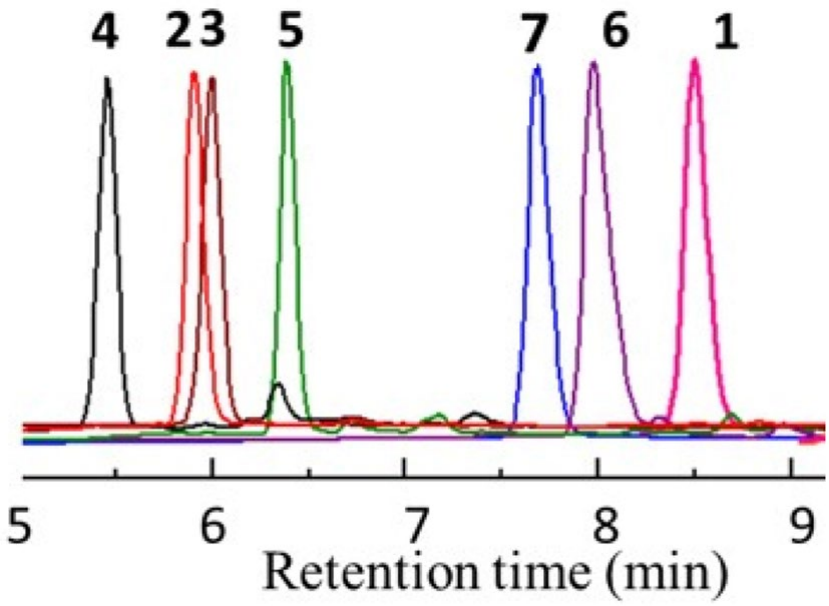
Reverse phase-high performance liquid chromatography (RP-HPLC) traces of macrocycle 1–7. Hydrophobicity increases from 4 to 1. (The mobile phase used was acetonitrile and water with 0.1% formic acid. The compounds were eluted with a solvent gradient of 5–95% of acetonitrile in water over 15 min).

**Figure 4:**
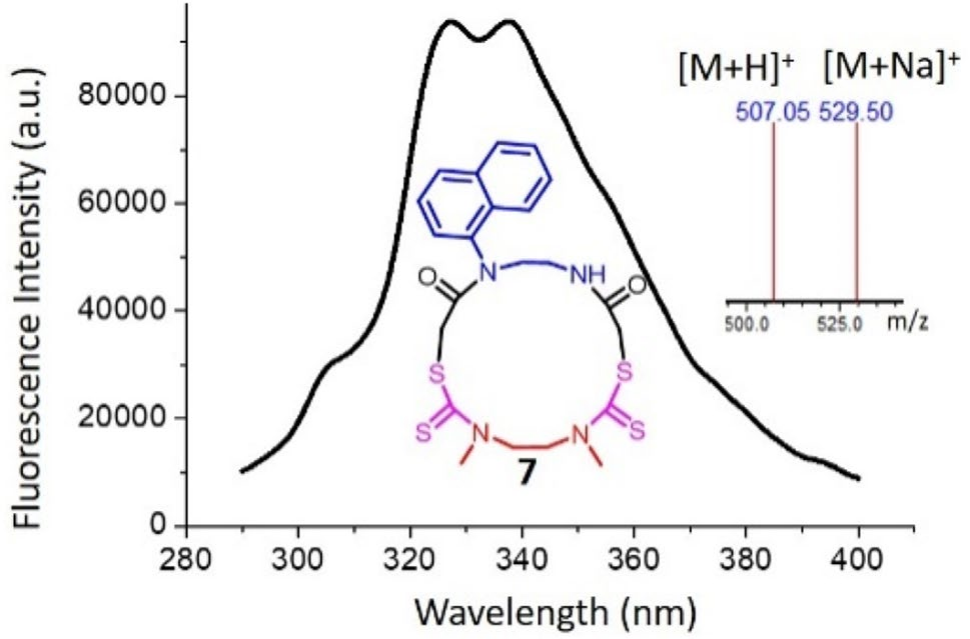
Structure and fluorescence spectrum of macrocycle 7 at 10 μM concentration in methanol upon excitation at 278 nm wavelength. The MS spectrum of the compound is given in the inset.

All the synthesized macrocycles were characterized by Liquid Chromatography-Mass Spectrometry (LC–MS), High Resolution Mass spectrometry (HRMS), ^1^H Nuclear Magnetic Resonance (NMR), ^13^C NMR and Infrared Spectroscopy (IR) (Figs. S12–S39). The characterization of a model macrocycle 1 is given in Fig. 2. The exact mass of the product is 492.17 Da, and the observed peak of 493.35 Da and 515.50 Da in the mass spectrum are assigned as its [M + H]^+^ and [M + Na]^+^, respectively (Fig. 2). The protons corresponding to the structure of the macrocycle 1 as well as the two starting materials have been assigned in the ^1^H NMR spectra as represented in Fig. 2. One unique advantage of this synthetic strategy is the possibility of synthesis and its purification in less than 90 minutes. The difference in property of the macrocycle is well evident from the Reverse Phase-High Performance Liquid Chromatography (RP-HPLC) by the change in retention time (Fig. 3). This is attributed to the change in hydrophobicity of the synthesized macrocycles. It was observed that macrocycle 1 is highly nonpolar due to two butyl groups while macrocycle 4 is more polar due to four –NH groups in it. Thus, the water solubility is also enhanced from macrocycle 1 towards 4, which is beneficial for various biological applications. The mass corresponding to each of the macrocycles was obtained in positive mode of the mass spectrum with [M + H]^+^ peak (Table 1). Macrocycle with a naphthyl group (7, Fig. 4) was also synthesized to utilize for photophysical studies. Dissimilar to others, 7 possesses three functional groups such as naphthyl, hydrogen and methyl, as the diamine used for chloroacetylation has two amine centres (naphthyl and hydrogen). This macrocycle was synthesized from a chloroacetylated diamide of *N*-naphthyl ethylene diamine. This macrocycle showed fluorescence when excited at 280 nm and Fig. 4 shows the fluorescence spectrum of 10 µM solution of macrocycle 7 along with the MS in the inset.

**Table 1:** Structural and characteristics details of the macrocycle.

Macrocycle	R	R′	n	n′	No. of atoms in the ring	Yield (%)	Calculated [M + H]^+^(Da)	Observed [M + H]^+^(Da)
1			2	2	16	61.3	493.17	493.35
2			2	2	16	24.1	409.08	409.26
3			2	3	17	78.5	395.06	395.60
4			3	3	18	85.3	381.25	381.65
5			3	6	21	74.7	423.09	423.60
6			3	2	17	69.4	547.12	547.21
7			2	2	16	84.6	507.09	507.05

R and R′ are the pendant functional group on macrocycle and n and n′ are the number of –CH_2_ groups on the diamine chain as shown in Fig. 1.

The significant factor which holds out from the scalable synthesis of macrocycles is the concentration of the starting materials in the reaction. As mentioned above, most macrocyclization reactions are carried out in dilute conditions to increase the probability of macrocycle formation. To synthesize in an industrially desired quantity, large volume of solvent is needed for dilute conditions. Hence, a technique that can satisfy the minimum solvent requirement is still in great demand. When many highly efficient macrocycles have to pay the cost of concentration in the micromolar range, and even in nanomolar range^13^, the concentration used here is about 0.2–0.4 M, which means only 500 μl of solvent is used for 50 mg of the starting material. The high concentration helped not only in less solvent usage but also in high yield and scalability. Subsequently, to make the macrocycle in gram scale, only 10–15 ml of the solvent is sufficient, which is highly acceptable. In this view, we synthesized 1.85 g of pure macrocycle by using 15 ml of the solvent (Fig. S40).

### Bioactive properties

**Figure 5:**
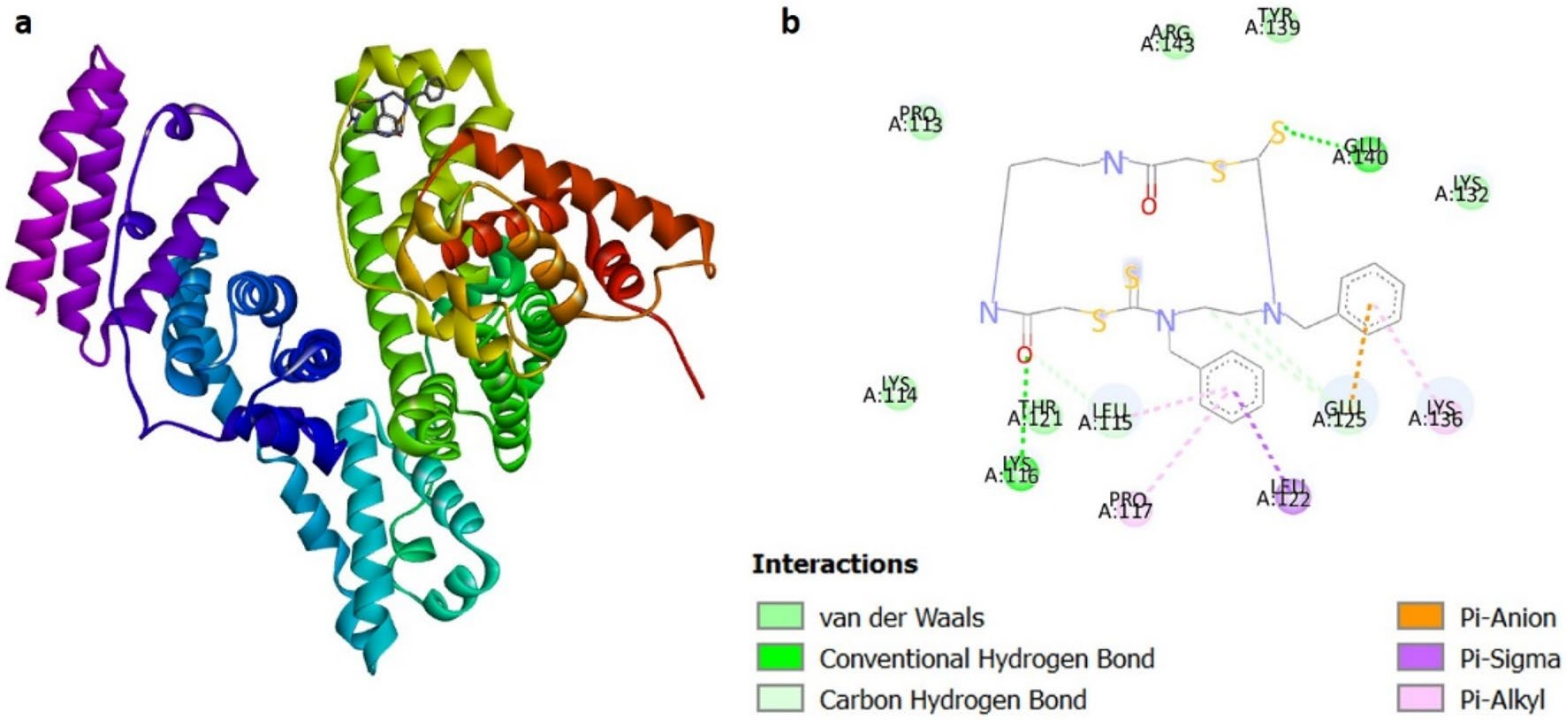
(**a**) The best-docked conformation of the macrocycle 6 with the BSA. (**b**) 2D diagram of amino acid interaction of BSA complexed with macrocycle 6. These figures were created using BIOVIA Discovery Studio Visualizer 2021 (https://www.3ds.com/products-services/biovia/products/molecular-modeling-simulation/biovia-discovery-studio/visualization/).

Druggability is an important parameter for the studies, and the Rule of Five is a set of guidelines that predicts the druggability of synthetic drugs by calculating their physico-chemical properties^8^. There are many potential macrocyclic drugs that show deviations from the rule, but still show promising activity. In 2014, Whitty et al. put forth guidelines for the synthetic macrocycles to produce a better drug with good bioavailability by reviewing many macrocyclic drugs^26^. We compared the properties of our synthesized macrocycles with the guidelines and observed that all the macrocycles are either following the conventional drug criteria or the advanced criteria for the macrocyclic drug (Table S2). This data brought up the exploratory hint that the macrocycles are druggable. The essential requirement of any molecule to act as a drug is to interact with a protein along with the prerequisites of its physicochemical properties. Here, we have selected a model protein serum albumin, the most abundant protein in the blood plasma and thus is the carrier for many important drugs^27^. The drug molecules bind with the protein and are carried to the target site through the bloodstream. Bovine Serum Albumin (BSA) was used for this study due to its 76% structural similarity with Human Serum Albumin (HSA), the easy availability and low cost. Molecular docking is a key tool to study the interaction of the protein with small molecules theoretically^28^. Hence, the docking studies were carried out with BSA and all the macrocycles, which showed that 6 has the maximum binding with the lowest binding energy (Table S3). The orientation of the macrocycles bound to the protein and the 2D interaction plots are shown in Fig. 5 and Suppl Figs. S41–S46. The results are compared with a standard molecule, a well-reputed drug—Ibuprofen. The binding energy of Ibuprofen in the similar range of the synthesized macrocycle directs it to the horizon of drug discovery.

**Figure 6:**
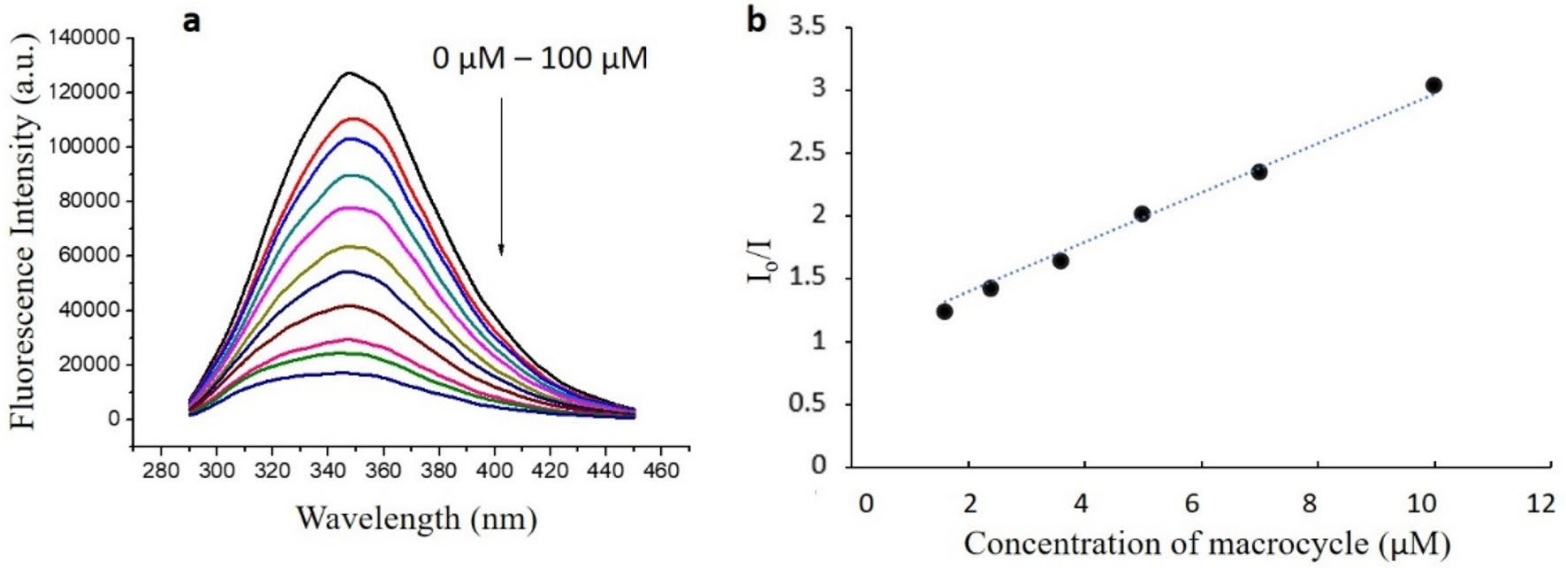
(**a**) Fluorescence quenching of BSA by macrocycle 6. To 2 ml of 10 µM BSA solution, 2–100 µl of macrocycle solution in DMSO (2 mM) was added at 25 °C and the excitation wavelength was 280 nm. (**b**) Stern–Volmer plot for the calculation of binding constant. I_o_ and I are fluorescence intensity at 0 µM and the given concentration of the macrocycle.

To validate the result experimentally, the interaction between the macrocycles and the BSA was studied by fluorescence spectroscopy by exploiting the intrinsic fluorescence of BSA by way of tryptophan and tyrosine residues^29^. It is well established that when there is an interaction between a molecule and BSA, the fluorescence intensity of the BSA changes due to the changes in conformation^30^. For the fluorescence studies, 10 μM solution of BSA was prepared in a phosphate buffer of pH 7.4. The macrocycles were dissolved in DMSO to make the stock solution concentration 2 mM. 0–100 μM solution was added in 10 μM BSA to study the fluorescence. Quenching of fluorescence was observed for all macrocycles indicating the interaction of the macrocycles with BSA (Fig. 6a, Figs. S47–S52), and the binding constant for each case was calculated from the Stern–Volmer Plot (Fig. 6b). The relative strength of binding of different macrocycles was obtained from the binding energy calculated by the molecular docking method (Table S3). The higher negative binding energy corresponds to the higher binding of the molecule with the protein, and this has further been confirmed experimentally through fluorescence study. The binding constant calculated through the fluorescence quenching study is in relative concurrence with the binding energy values from the molecular docking study, with few exceptions. Thus, the preliminary docking studies are helpful to predict the functional groups and ring size, which could give better binding with the protein. As a positive control, the docking with the well-known drug ibuprofen was also calculated, and the binding constant with BSA protein is already reported^31^. The change in functional groups and ring size rationally resulted in bringing the binding constant of the macrocycle to the range of the binding constant of Ibuprofen (Table 2). An about 25-fold increase in the binding constant was made from macrocycle 2 (binding constant: (1.66 ± 0.32) × 10^3^ M^−1^) to macrocycle 6 (binding constant: (39.43 ± 1.02) × 10^3^ M^−1^) by the systematic change in the functional group and ring size (Table 2). Macrocycle 6 showed the highest binding constant, followed by 7 with a binding constant of (19.67 ± 0.86) × 10^3^ M^−1^. The increase in the binding constant is allocated by the phenyl groups due to π–π interaction and hydrogen bonding due to –NH groups. The macrocycle with four –NH groups, 5 also showed higher binding, which is likely because of the formation of hydrogen bonding with the residues. Hence, the synthesized macrocycles have been proven to interact with model protein and thus can be extended to study the interaction with other target proteins in the future.

**Table 2:**
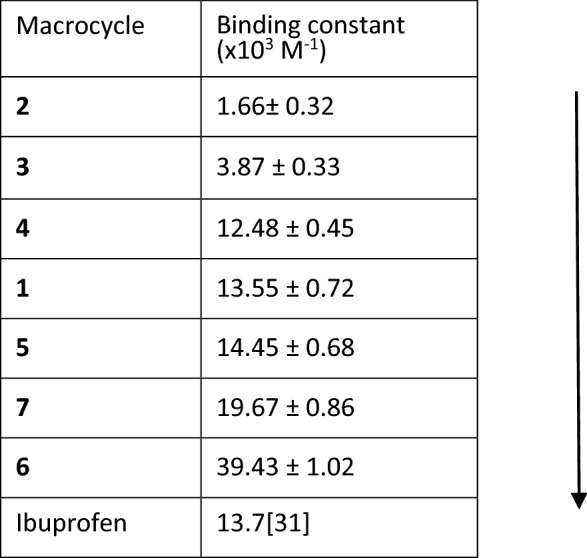
The binding constant of the synthesized macrocycles and the known drug ibuprofen.

Twenty-five times increase in the biding constant was obtained from macrocycle 2 to 6.

## Conclusion

In conclusion, a scalable, fast and efficient two-step synthetic strategy has been established to introduce a new macrocycle class. To enhance the activity, a biologically important entity, dithiocarbamate, has been incorporated in the backbone of the macrocycle for the first time without the aid of metal. This class of macrocycle holds the property of tunable functional groups and extends to different ring sizes. This strategy accomplished the synthesis of macrocycle at gram-scale in high concentrations (0.2–0.4 M) within 90 min with good yields. The druggability studies of the macrocycles and the theoretical and experimental studies on the binding of the macrocycles with the protein provide good support on the potential of the synthesized macrocycles towards drug discovery. As the dithiocarbamates are a versatile entity for biomedical application, by supplementing the advantages of macrocyclic scaffolds, we are currently exploring the therapeutical properties of the synthesized macrocycles.

## Experimental section

### Synthesis of N,N′-dibutyl ethylenediamine

To the solution of 1,2-dichloroethane (240 µl, 3.03 mmol, 1 eq.) in *N,N*-dimethyl formamide (DMF) (9 ml), butylamine (199 µl, 6.06 mmol, 2 eq.) was added and the mixture was kept at 90 °C and stirred for 3 h. After the completion of the reaction, the reaction mixture was extracted by water and ethyl acetate in a 1:4 ratio and the ethyl acetate layer were dried over anhydrous Na_2_SO_4_. The product was isolated from the ethyl acetate under reduced pressure. The synthesized compound was directly taken for the next step without purification (Yield = 78.4%).

### Synthesis of chloroacetylated diamides

Chloroacetylated diamides were synthesized from the reaction of diamines (1 mmol, 1 eq.) and chloroacetyl chloride (4 mmol, 4 eq.) in the presence of a base triethylamine (4 mmol, 4 eq.) in the solvent DCM (10 ml) under room temperature. The reaction was monitored by TLC in the 7:3 system of ethyl acetate and hexane and visualized under UV light. After completion of reaction (15 min), the excess chloroacetyl chloride was quenched by adding sodium bicarbonate solution until the evolution of CO_2_ ceased. The reaction mixture was extracted by DCM and water and the organic layer was passed through anhydrous Na_2_SO_4_. The solvent was removed under low pressure and the product was obtained under high purity (Yield = 94%).

### Synthesis of macrocycles

Chloroacetylated diamide (0.2 mmol, 1 eq.) was taken in 0.5 ml of Polyethylene glycol (PEG)-200 and a diamine (0.24 mmol, 1.2 eq.) and CS_2_ (1.6 mmol, 8 eq.) was added to this and stirred for 15 min under room temperature. The progress of the reaction was monitored by TLC in 7:3 ethyl acetate and hexane solution and visualized under UV light and iodine. After the completion of the reaction, the reaction mixture was extracted by using water and ethyl acetate. Both the organic layers were passed through anhydrous Na_2_SO_4_. The solvent was removed under low pressure and the product was taken for further purification.

### Fluorescence spectroscopy for protein interaction studies

Fluorescence was recorded on Perkin Elmer FL 6500. All fluorescence spectra are recorded at 25 °C with an excitation wavelength of 280 nm and a slit width of 5 nm for excitation and emission. 10 μM BSA was prepared by dissolving 6.6 mg of BSA in 10 ml of phosphate buffer of pH 7.4. The macrocycles were dissolved in DMSO to make the stock solution of 2 mM concentration. 2 ml of the BSA control solution was taken in the fluorescence cuvette to take the emission at zero concentration of the compound. The titration was carried out by varying the concentration of macrocycle from 2 to 100 μM. An equilibration time of 3 min is given for each measurement after the addition of the solution. Binding constants were estimated from the Stern–Volmer plot by plotting I_o_/I vs concentration of the macrocycle. The slope of the graph was the attributed as the binding constant.

### Molecular docking studies

The software comprising of Autodock Tools4.2.6^32^, Autodock Vina_1_1_2^33^, was used to perform molecular docking of BSA with the macrocycles. The crystal structure of BSA (ID-4F5S) was downloaded from Protein Data Bank and the macrocycles were energy minimized by ArgusLab 4.0.1. By using the MM-UFF method. Chain A of BSA was used for docking by removing chain B and water molecules. Polar hydrogens and partial Kollmann charges were added by merging the nonpolar hydrogens with BSA. The grid file used was of the dimension center x, y, z = 9.93, 20.81, 99.21 and of size x, y, z = 93.95. 61.72, 84.48. The output of the results was estimated using the Lamarckian genetic algorithm. PyMol was used to get the pdb format of the docked structure and Discovery Studio Visualizer was used for the visualization of the docked structure.

### HPLC separation and LC–MS analysis

The purification by HPLC is performed on Shimadzu HPLC-20AP instrument by using the same solvent system as LC–MS. Solvents used were water with 0.1% formic acid (solvent A) and acetonitrile with 0.1% formic acid (solvent B). Compounds were eluted at a flow rate of 19 ml/min with a gradient of 20%, 60%, 75%, 90% and 20% of acetonitrile over 26 min. LC–MS experiments were carried out on a Shimadzu LC–MS-8045 with a Sprite TARGA C18 column (40 × 2.1 mm, 5 µm) monitoring at 254 nm (unless not specified) with positive mode for mass detection. Solvents for LC–MS were water with 0.1% formic acid (solvent A) and acetonitrile with 0.1% formic acid (solvent B). Compounds were eluted at a flow rate of 0.5 ml/min with a gradient of 5%, 60%, 90% and again 5% of acetonitrile over the time of 15 min.

### HRMS, NMR and IR

HRMS was recorded in Waters ACQUITY H-CLASS + UPLC/XevoG2 XS QTOF instrument. ^1^H NMR spectra were recorded on an INOVA-400 spectrometer and Bruker AV III 500 MHz. The data were analyzed by MestReNova (version 8.1.1). ^1^H NMR shifts are reported in units of ppm relative to tetramethyl silane. The data are presented in the order: chemical shift, peak multiplicity (s = singlet, d = doublet, t = triplet, m = multiplet) and proton number. Fourier Transformed IR Spectroscopy (FT-IR) was recorded in Shimadzu IR Tracer 100 in Attenuated Total Reflection (ATR) method.

## Supplementary Information

Supplementary Information.

## References

[1] Guo Z (2019). Rapamycin-inspired macrocycles with new target specificity. Nat. Chem..

[2] Ermert P (2017). Design, properties and recent application of macrocycles in medicinal chemistry. Chimia (Aarau).

[3] Shen K, Yang X, Cheng Y, Zhu C (2012). A highly selective ratiometric fluorescent chemosensor for Zn^2+^ ion based on a polyimine macrocycle. Tetrahedron.

[4] Iyoda M, Yamakawa J, Rahman MJ (2011). Conjugated macrocycles: Concepts and applications. Angew. Chem. Int. Ed..

[5] Kim DJ (2017). Redox-active macrocycles for organic rechargeable batteries. J. Am. Chem. Soc..

[6] Driggers EM, Hale SP, Lee J, Terrett NK (2008). The exploration of macrocycles for drug discovery—An underexploited structural class. Nat. Rev. Drug Discov..

[7] Dinsmore CJ (2001). Conformational restriction of flexible ligands guided by the transferred NOE experiment: Potent macrocyclic inhibitors of farnesyltransferase [25]. J. Am. Chem. Soc..

[8] Lipinski CA, Lombardo F, Dominy BW, Feeney PJ (1997). Experimental and computational approaches to estimate solubility and permeability in drug discovery and development settings. Adv. Drug Deliv. Rev..

[9] Otsuka H (2013). Reorganization of polymer structures based on dynamic covalent chemistry: Polymer reactions by dynamic covalent exchanges of alkoxyamine units. Polym. J..

[10] Bédard A-C, Collins SK (2011). Phase separation as a strategy toward controlling dilution effects in macrocyclic Glaser-Hay couplings. J. Am. Chem. Soc..

[11] Fan Q (2017). On-surface pseudo-high-dilution synthesis of macrocycles: Principle and mechanism. ACS Nano.

[12] Martí-Centelles V, Burguete MI, Luis SV (2016). Macrocycle synthesis by chloride-templated amide bond formation. J. Org. Chem..

[13] Collins JC, James K (2012). Emac—A comparative index for the assessment of macrocyclization efficiency. MedChemComm.

[14] Ding Y (2018). Synthesis and biological evaluation of dithiocarbamate esters of parthenolide as potential anti-acute myelogenous leukaemia agents. J. Enzyme Inhib. Med. Chem..

[15] Stasevich MV (2020). 9, 10-Anthraquinone Dithiocarbamates as potential pharmaceutical substances with pleiotropic actions: Computerised prediction of biological activity and experimental validation. Pharm. Chem. J..

[16] Baghershiroudi M, Safa KD, Adibkia K, Lotfipour F (2018). Synthesis and antibacterial evaluation of new sulfanyltetrazole derivatives bearing piperidine dithiocarbamate moiety. Synth. Commun..

[17] de Oliveira JWF, Rocha HAO, de Medeiros WMTQ, Silva MS (2019). Application of dithiocarbamates as potential new antitrypanosomatids-drugs: Approach chemistry, functional and biological. Molecules.

[18] Ekennia AC, Onwudiwe DC, Osowole AA, Olasunkanmi LO, Ebenso EE (2016). Synthesis, biological, and quantum chemical studies of Zn (II) and Ni (II) mixed-ligand complexes derived from N, N-disubstituted dithiocarbamate and benzoic acid. J. Chem..

[19] Pal DS, Mondal DK, Datta R (2015). Identification of metal dithiocarbamates as a novel class of antileishmanial agents. Antimicrob. Agents Chemother..

[20] Skrott Z (2017). Alcohol-abuse drug disulfiram targets cancer via p97 segregase adaptor NPL4. Nature.

[21] Kadu R, Roy H, Singh VK (2015). Diphenyltin (IV) dithiocarbamate macrocyclic scaffolds as potent apoptosis inducers for human cancer HEP 3B and IMR 32 cells: Synthesis, spectral characterisation, density functional theory study and in vitro cytotoxicity. Appl. Organomet. Chem..

[22] Beer PD (2001). Self-assembled dithiocarbamate–copper (ii) macrocycles for electrochemical anion recognition. Chem. Commun..

[23] Torres-Huerta A, Rodriguez-Molina B, Höpfl H, Garcia-Garibay MA (2014). Synthesis and solid-state characterization of self-assembled macrocyclic molecular rotors of bis (dithiocarbamate) ligands with diorganotin (IV). Organometallics.

[24] Steinhardt J, Krijn J, Leidy JG (1971). Differences between bovine and human serum albumins. Binding isotherms, optical rotatory dispersion, viscosity, hydrogen ion titration, and fluorescence effects. Biochemistry.

[25] Vafaeezadeh M, Hashemi MM (2015). Polyethylene glycol (PEG) as a green solvent for carbon-carbon bond formation reactions. J. Mol. Liq..

[26] Villar EA (2014). How proteins bind macrocycles. Nat. Chem. Biol..

[27] Kratz F, Müller-Driver R, Hofmann I, Drevs J, Unger C (2000). A novel macromolecular prodrug concept exploiting endogenous serum albumin as a drug carrier for cancer chemotherapy. J. Med. Chem..

[28] Kitchen DB, Decornez H, Furr JR, Bajorath J (2004). Docking and scoring in virtual screening for drug discovery: methods and applications. Nat. Rev. Drug Discov..

[29] Bobone S, van de Weert M, Stella L (2014). A reassessment of synchronous fluorescence in the separation of Trp and Tyr contributions in protein emission and in the determination of conformational changes. J. Mol. Struct..

[30] Eftink MR, Ghiron CA (1981). Fluorescence quenching studies with proteins. Anal. Biochem..

[31] Tanaka M, Asahi Y, Masuda S, Ota T (1991). Binding position of Ibuprofen with bovine serum albumin determined by measuring nuclear magnetic resonance relaxation time. Chem. Pharm. Bull..

[32] Morris GM (2009). AutoDock4 and AutoDockTools4: Automated docking with selective receptor flexibility. J. Comput. Chem..

[33] Trott O, Olson A (2010). AutoDock Vina: Improving the speed and accuracy of docking with a new scoring function, efficient optimization, and multithreading. J. Comput. Chem..

