# Supplementary material for: Design, synthesis and bioactive properties of a class of macrocycles with tunable functional groups and ring size
Source: Sci Rep. 2022 Mar 21;12:4815. doi: 10.1038/s41598-022-08775-z (PMC8938452; doi:10.1038/s41598-022-08775-z)
Supplement: Supplementary file 1 — Supplementary Information. [file 41598_2022_8775_MOESM1_ESM.pdf]

**Supporting Information**

# Design, synthesis and bioactive properties of a class of macrocycles with tunable functional groups and ring size

**Liya Thurakkal, Pandurangan Nanjan, Mintu Porel***

Department of Chemistry

Environmental Sciences and Sustainable Engineering Center

Indian Institute of Technology Palakkad, Kerala 678577, India

1. Materials and Methods

All the chemicals were purchased from Sigma Aldrich, Alfa Aeser, Spectrochem and TCI and used without further purification. LC-MS experiments were carried out on a Shimadzu LC-MS-8045 with a Sprite TARGA C18 column (40 × 2.1 mm, 5 µm) monitoring at 254 nm (unless not specified) with positive mode for mass detection. Solvents for LC-MS were water with 0.1% formic acid (solvent A) and acetonitrile with 0.1% formic acid (solvent B). Compounds were eluted at a flow rate of 0.5 ml/min with a gradient of 5%, 60%, 90% and again 5% of acetonitrile over the time of 15 minutes. The purification by HPLC is performed on Shimadzu HPLC-20AP instrument by using the same solvent system of that of LC-MS. Compounds were eluted at a flow rate of 19ml/min with a gradient of 20%, 60%, 75%, 90% and 20% of acetonitrile over 26 minutes. $^1$H NMR spectra were recorded on INOVA-400 spectrometer and and Bruker AV III 500 MHz. The data were analyzed by MestReNova (version 8.1.1) (https://mestrelab.com/software/mnova/nmr/). $^1$H NMR shifts are reported in units of ppm relative to tetramethyl silane. The data are presented in the order: chemical shift, peak multiplicity (s=singlet, d=doublet, t=triplet, m=multiplet) and proton number. Fluorescence was recorded on Perkin Elmer FL 6500. All fluorescence spectra are recorded at 25$^o$C with an excitation wavelength of 280 nm and slit width of 5 nm for excitation and emission. The fluorescence spectra were plotted in OriginPro 8.5.1 (https://www.originlab.com/). HRMS was measured in Waters ACQUITY H-CLASS + UPLC/XevoG2 XS QTOF instrument. Fourier Transformed IR Spectroscopy (FT-IR) was recorded in Shimadzu IR Tracer 100 in Attenuated Total Reflection (ATR) method and spectra were plotted in OriginPro 8.5.1. The software comprising of Autodock Tools[1], Autodock Vina[2] was used to perform molecular docking of BSA with the macrocycles. The crystal structure of BSA (ID-4F5S) was downloaded from Protein Data Bank and the macrocycles were energy minimized by ArgusLab 4.0.1. by using MM-UFF method[3]. Chain A of BSA was used for docking by removing chain B and water molecules. Polar hydrogens and partial Kollmann charges were added by merging the nonpolar hydrogens to BSA. The grid file used was of the dimension center x, y, z = 9.93, 20.81, 99.21 and size x, y, z= 93.95. 61.72, 84.48. The output of the results was estimated using Lamarckian genetic algorithm. PyMol was used to get the pdb format of the docked structure and BIOVIA Discovery Studio Visualizer 2021 was used for the visualization of the docked structure (https://www.3ds.com/products-services/biovia/products/molecular-modeling-simulation/biovia-discovery-studio/visualization).

2. Solvent screening

To study the effect of solvent, the synthesis of macrocycle was carried out in different solvents and the yield for the macrocycle obtained was calculated and are tabulated below. Synthesis of macrocycle **3** has been taken as an example for the study. It was observed that the yield was higher when the reaction was carried out in PEG-200 as the solvent followed by glycerol.

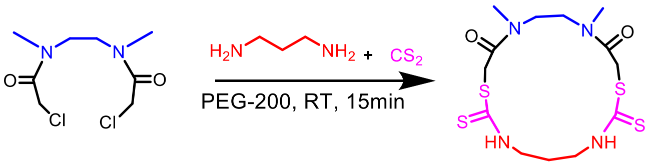


**Figure S1.** The reaction of synthesis of macrocycle **3,** employed for solvent screening

**Table S1.** Solvent screening for macrocyclization reaction.

| Sl. No | Solvent | Yield |
|---|---|---|
| 1 | Dimethyl formamide (DMF) | 5.1% |
| 2 | Acetonitrile | 17.8% |
| 3 | Ethanol | 8.1% |
| 4 | Tetrahydrofuran (THF) | 5.3% |
| 5 | Polyethylene glycol-200 (PEG200) | 80% |
| 6 | Water | 5.7% |
| 7 | Glycerol | 38% |
| 8 | Dichloromethane (DCM) | 2.4% |

3. Kinetics

Kinetics study of macrocyclization reaction was carried out with the reaction of chloroacetylated N, N'-dimethyl ethylene diamide with $CS_2$ and 1,3-propane diamine to produce macrocycle **3**. To monitor the kinetics of the reaction, samples were aliquoted from the reaction mixture at time t=0 min, 5 min, 10 min and 15 min. LCMS were recorded for each sample. From **Figure. S1**, it was observed that the reaction was completed within 15 minutes. At 15 minutes, the disappearance of starting material indicating the completion of the reaction.

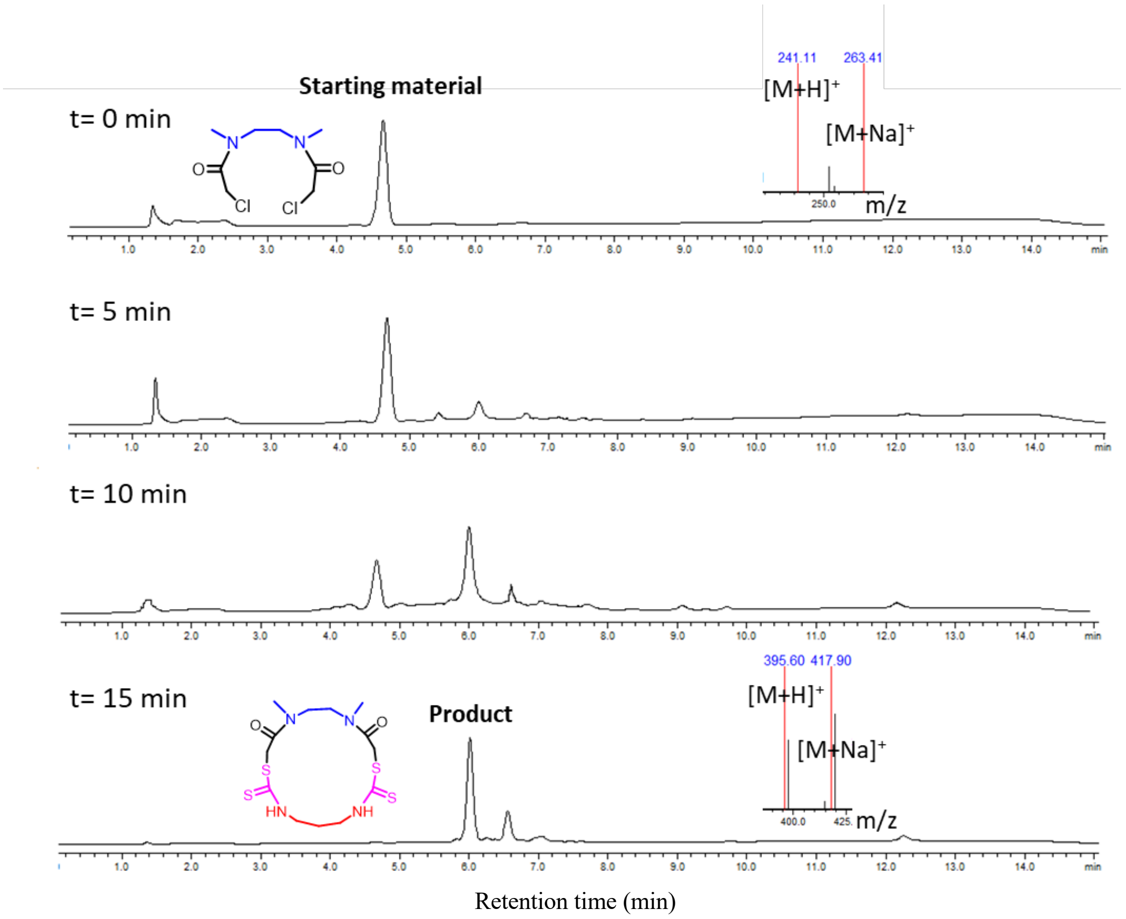


**Figure S2.** Kinetics study of the macrocyclization reaction (given is the synthesis of **3**)

4. Synthetic procedures and characterization

4.1 N,N'-dibutyl ethylenediamine

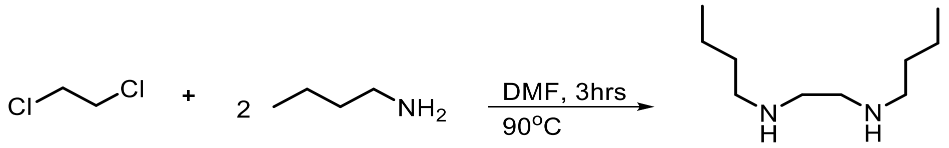


**Figure S3**. Synthesis of N,N'-dibutyl ethylenediamine. (DMF: N,N dimethyl formamide)

To the solution of 1,2-dichloroethane (240 µl, 3.03 mmol, 1eq.) in N,N-dimethyl formamide (DMF) (9 ml), butyl amine (199 µl, 6.06 mmol, 2 eq.) was added and the mixture was kept at 90°C and stirred for 3 hrs. After the completion of reaction, the reaction mixture was extracted by water and ethyl acetate in 1:4 ratio and the ethyl acetate layer were dried over anhydrous $Na_2SO_4$. The product was isolated from the ethyl acetate under reduced pressure. The synthesized compound was directly taken for the next step without purification (Yield = 78.4%).

*4.1.1. LCMS*

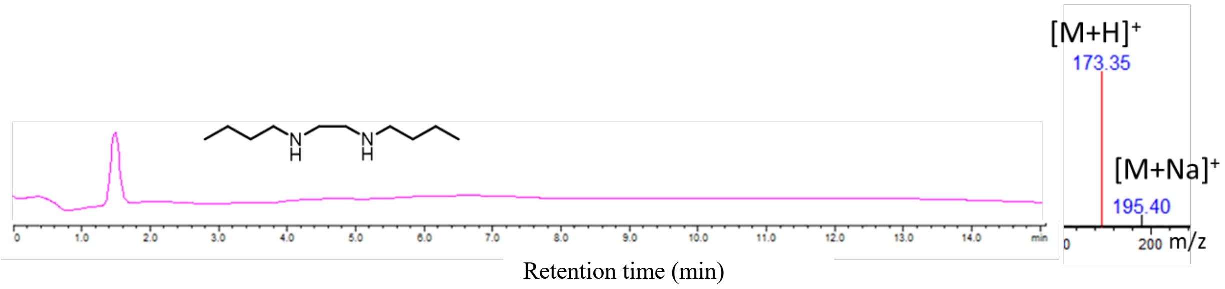


**Figure S4.** LCMS of N, N'- dibutyl ethylenediamine without any purification. LC-MS calculated $[M+H]^+$: 173.19 Da, observed $[M+H]^+$:173.35 Da, $[M+Na]^+$: 195.40 Da.

*4.1.2 $^1$H NMR*

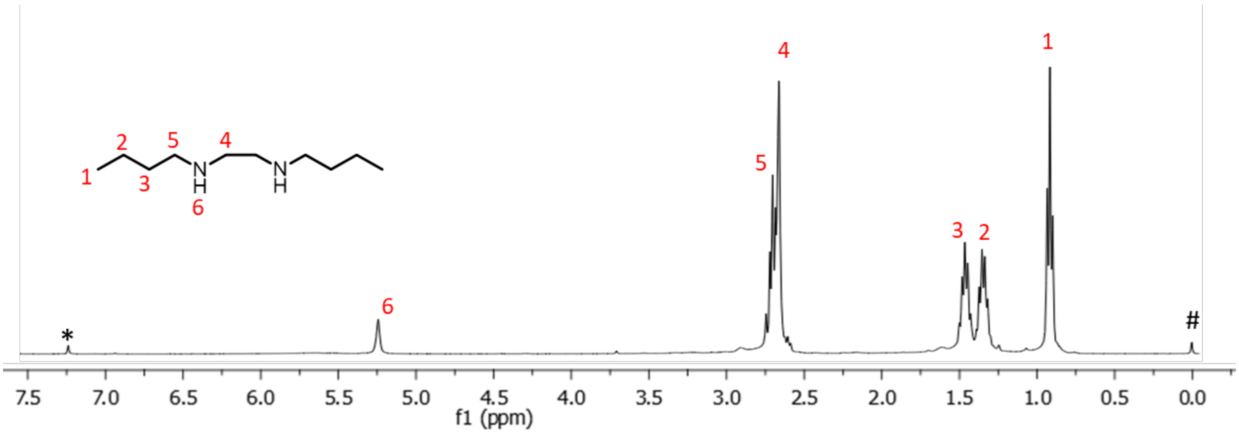


**Figure S5.** $^1$H NMR (400MHz, $CDCl_3$) spectrum of **d1**: δ (ppm) 0.90 (t, 3H), 1.345 (m, 4H), 1.474 (m, 4H), 2.682 (s, 4H), 2.764 (t, 4H). "#" and "*" represent the residual proton signal of internal standard tetramethylsilane and $CDCl_3$ respectively.

4.2 Chloroacetylated diamides

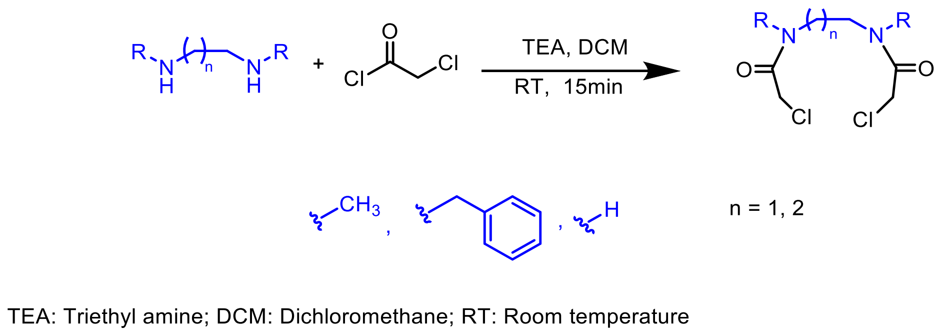


**Figure S6.** Synthesis of chloroacetylated diamides

Chloroacetylated diamides were synthesized from the reaction of diamines (1 mmol, 1 eq.) and chloroacetyl chloride (4 mmol, 4 eq.) in presence of a base triethylamine (4 mmol, 4 eq.) in the solvent DCM (10 ml) under room temperature. The reaction was monitored by TLC in 7:3 system of ethyl acetate and hexane and visualized under UV light. After completion of reaction (15 min), the excess chloroacetyl chloride was quenched by the addition of sodium bicarbonate

solution until the evolution of $CO_2$ ceased. The reaction mixture was extracted by DCM and water and the organic layer was passed through anhydrous $Na_2SO_4$. The solvent was removed under low pressure and the product was obtained under high purity (Yield = 94%).

### *4.2.1 Chloroacetylated N, N'-dimethyl ethylenediamine*

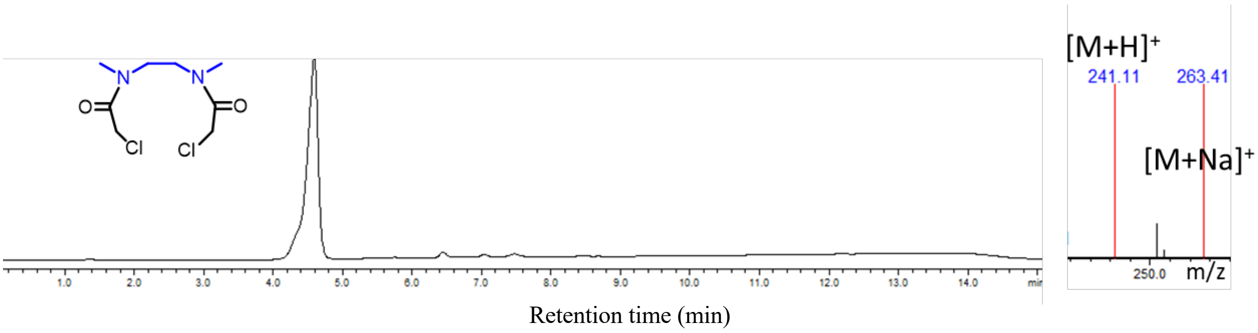


**Figure S7.** LCMS of chloroacetylated diamide of **c1**. LC-MS calculated $[M+H]^+$: 241.04 Da, observed $[M+H]^+$: 241.11 Da, $[M+Na]^+$: 263.41 Da

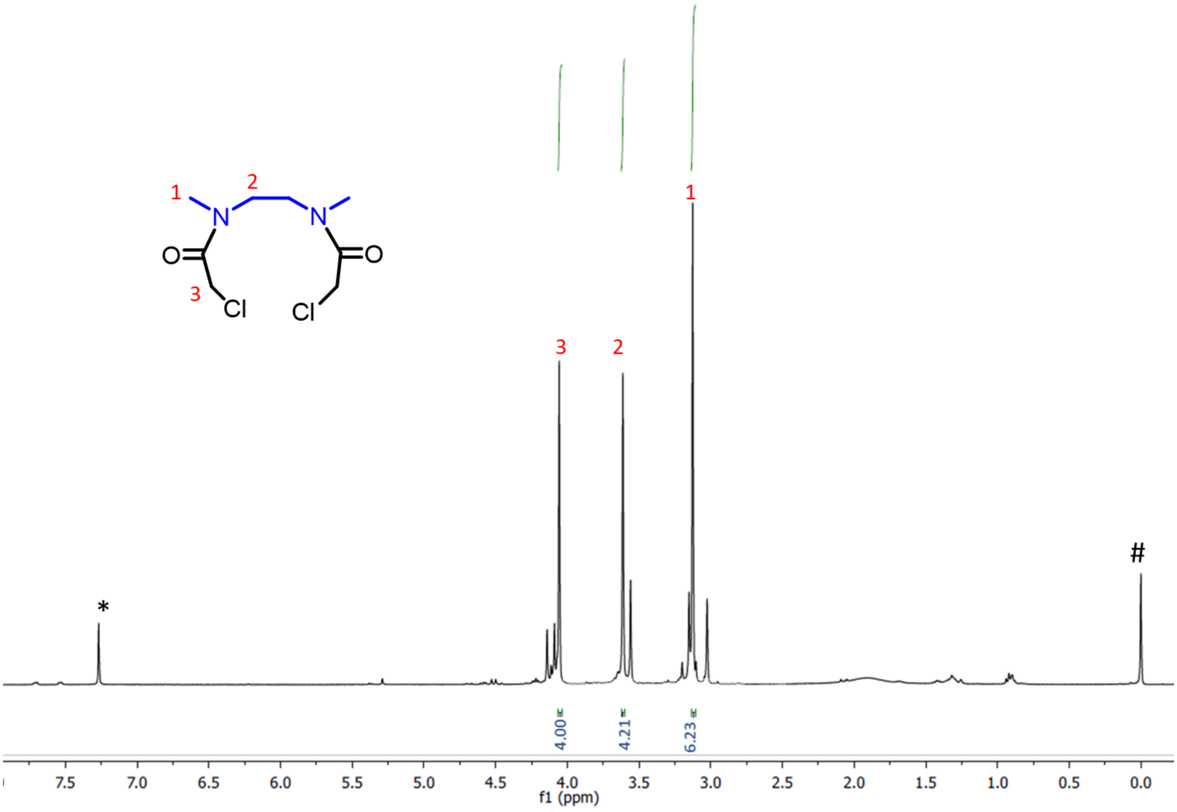


**Figure S8.** $^1H$ NMR (400MHz, $CDCl_3$) spectrum of **c1**: δ (ppm) 3.18 (s,6H), 3.62 (s, 4H), 4.12 (s,4H). "#" and "*" represent the residual proton of internal standard tetramethylsilane and $CDCl_3$ respectively.

### *4.2.2 Chloroacetylated 1, 3-propanediamine*

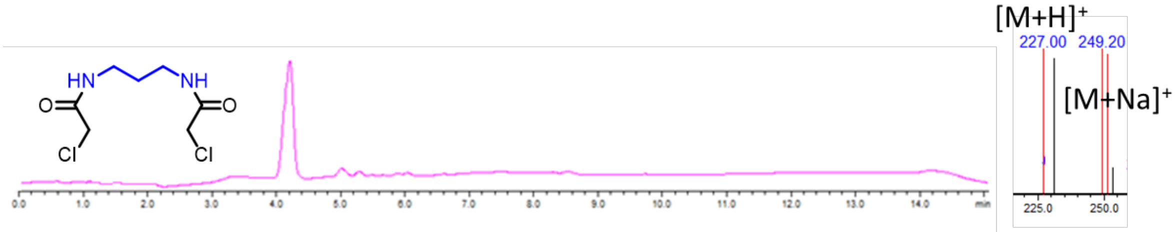


**Figure S9.** LCMS of chloroacetylated diamide of 1, 3-propane diamine (**c2**). LC was monitored in the detector of wavelength 210nm. LC-MS calculated $[M+H]^+$: 227.03 Da, observed $[M+H]^+$: 227.00 Da, $[M+Na]^+$: 249.20 Da.

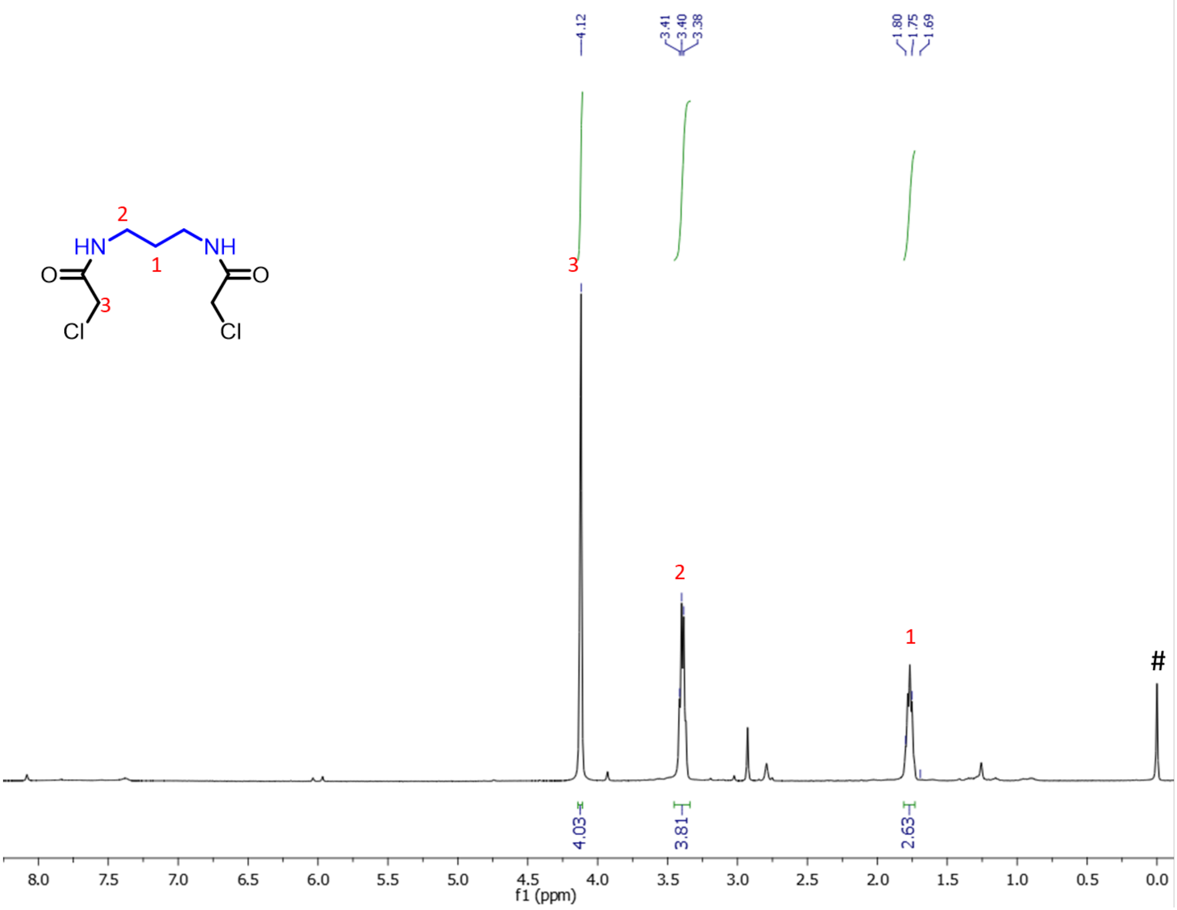


**Figure S10.** $^1$H NMR (400MHz, $CDCl_3$) spectrum of **c2**: δ (ppm) 1.76 (t, J=20Hz, 2H), 3.40 (t, J=6Hz, 4H), 4.11 (s, 4H). "#" represents the residual proton of internal standard tetramethylsilane

4.3 Macrocycles

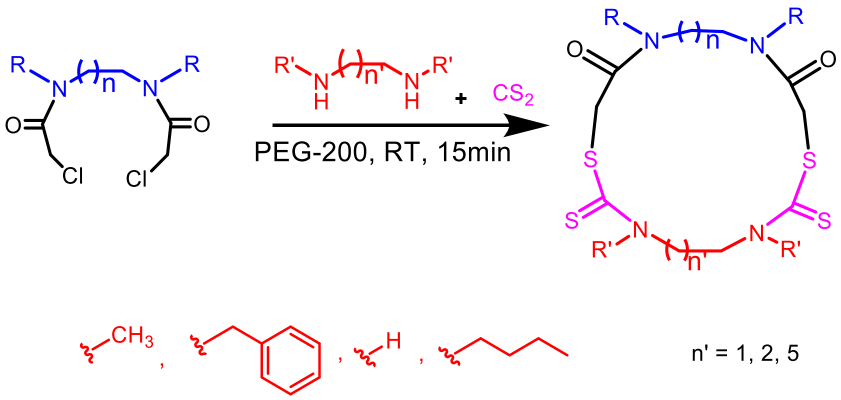


**Scheme S11.** Synthesis of macrocycle

Chloroacetylated diamide (0.2 mmol, 1 eq) was taken in 0.5 ml of Polyethylene glycol (PEG)-200 and a diamine (0.24 mmol, 1.2 eq.) and $CS_2$ (1.6 mmol, 8 eq.) was added to this and stirred for 15 minutes under room temperature. The progress of the reaction was monitored by TLC in 7:3 ethyl acetate and hexane solution and visualized under UV light and iodine. After completing the reaction, the reaction mixture was extracted by using water and ethyl acetate. The polymer obtained (majorly for macrocycle **6)** was removed during extraction. The organic layer was passed through anhydrous $Na_2SO_4$. The solvent was removed under low pressure and the product was taken for further purification. The purification of each macrocycle was carried out by column chromatography with silica gel 200-400 mesh. The pure product was eluted in the solvent ratio as given: Macrocycle **1**: 40% ethyl acetate in hexane, **2**: 5% methanol in ethyl acetate, **3**: 5% methanol in ethyl acetate, **4**: 10% methanol in ethyl acetate, **5**: 100% ethyl acetate, **6**: 50% ethyl acetate in hexane and **7**: 65% ethyl acetate in hexane.

*4.3.1 Macrocycle **1***

The macrocycle is synthesized by following the abovementioned procedure and purified by column chromatography isolated as colorless oil (yield: 61.3%). $^{1}$H NMR (400MHz, $CDCl_3$) spectrum of macrocycle **1**. δ (ppm) 0.93 (t, J= 5.0 Hz, 6H), 1.35 (m, J= 2.50 Hz, 4H), 1.49(m, J=2.50 Hz, 4H), 2.74 (t, J= 5.0 Hz, 4H), 3.61 (s, 4H), 3.66 (s, 8H), 3.73 (s, 6H). "#" and "*" represent the residual proton of internal standard tetramethyl silane and $CDCl_3$ respectively. $^{13}$C NMR (125 MHz, $CDCl_3$) δ 14.01, 20.31, 29.11, 36.42, 40.85,45.96, 47.38, 167.90 and 182.60. LC-MS calculated $[M+H]^+$: 493.17 Da, observed $[M+H]^+$: 493.25 Da, $[M+Na]^+$: 515.50 Da. HRMS (ESI) m/z calculated for $C_{20}H_{36}N_4O_2S_4$ [M+H]+ : 493.1721 Da; found [M+H]+ : 493.1554 Da. IR (ATR) $\tilde{\upsilon}$ = 1630 $cm^{-1}$ (C=O), 1119 $cm^{-1}$ (symmetric stretch, C=S) and 1070 $cm^{-1}$ (asymmetric stretch, C=S)

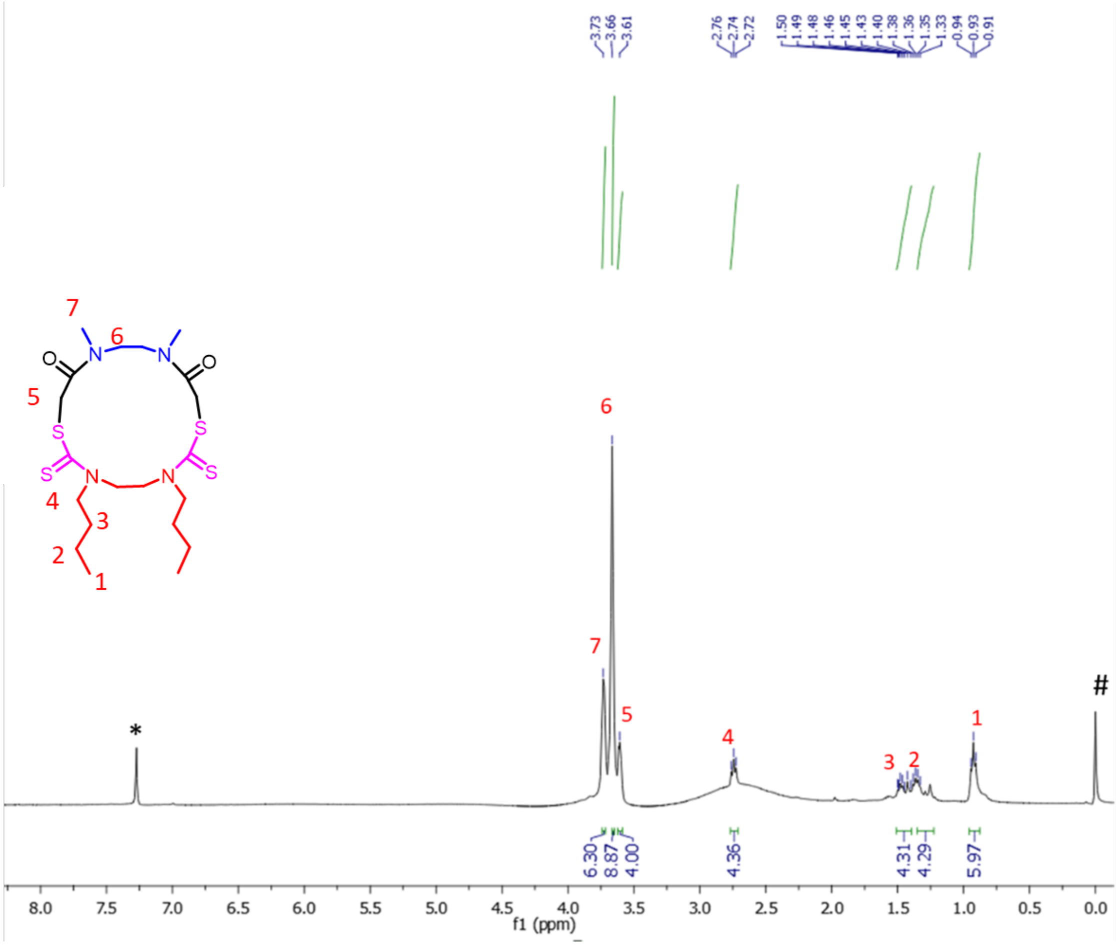


**Figure S12:** $^{1}$H-NMR (400MHz) of macrocycle **1** in $CDCl_3$

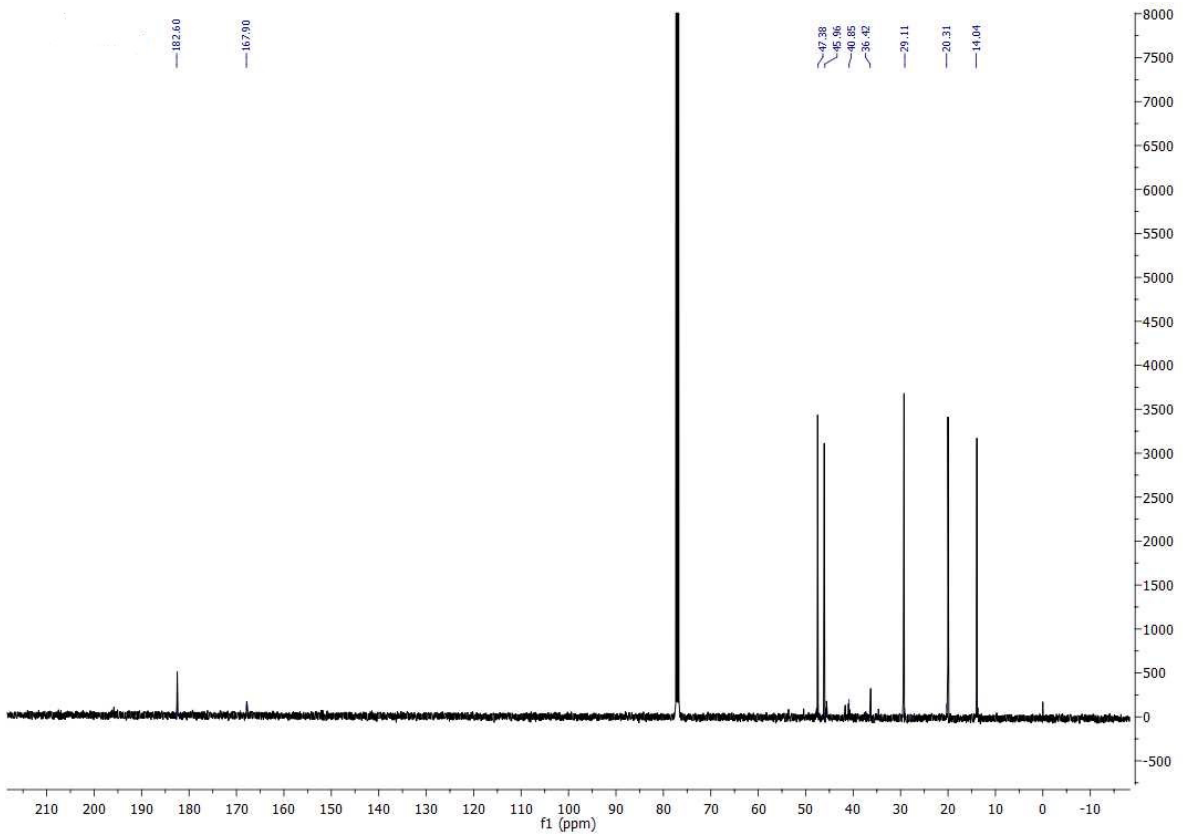


**Figure S13:** $^{13}$C-NMR of macrocycle **1** in $CDCl_3$

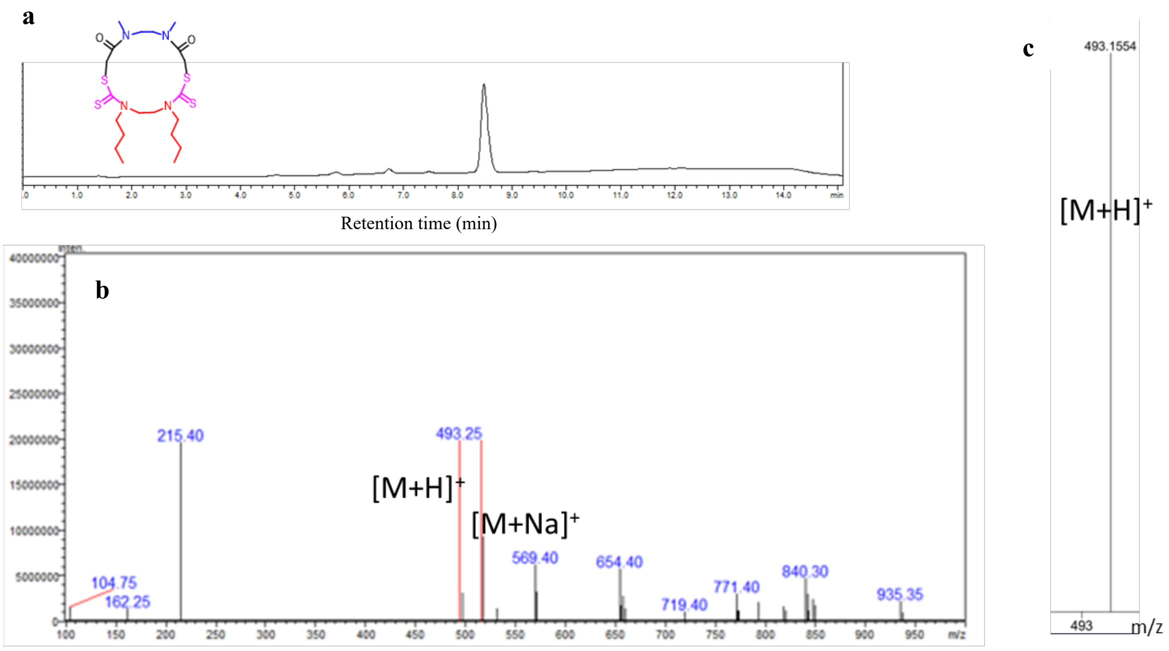


**Figure S14 a**: HPLC of macrocycle **1** eluted in acetonitrile and water gradient recorded at 254 nm. **b**: Full range MS spectrum in positive ion mode. **c**: HRMS with $[M+H]^+$ peak

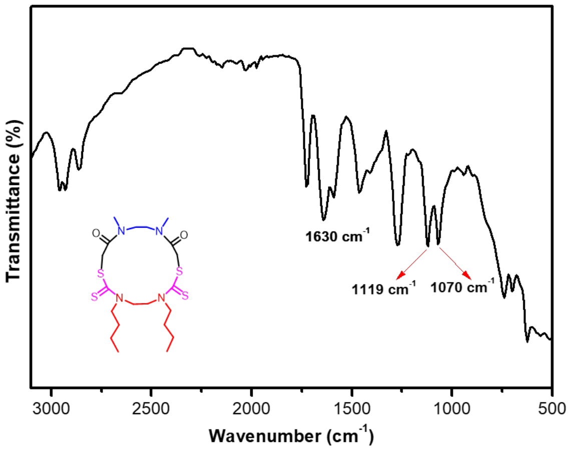


**Figure S15: .** FT-IR spectrum of macrocycle **1.**

*4.3.2 Macrocycle* ***2***

The macrocycle is synthesized by following the procedure given and purified by column chromatography, isolated as white solid (24.1%). $^1$H NMR (400MHz, DMSO-d6) spectrum of macrocycle **2**. δ (ppm) 3.40 (s, 12H), 3.43 (s, 4H), 3.512 (s, 8H). "*" represent the residual proton of DMSO-d6. $^{13}$C NMR (125 MHz, $CDCl_3$) δ 33.51, 37.17, 47.77, 48.88, 53.64, 169.40, 197.58. LC-MS calculated $[M+H]^+$: 409.08 Da, observed $[M+H]^+$: 409.26 Da, $[M+Na]^+$: 431.36 Da. HRMS (ESI) m/z calculated for $C_{14}H_{24}N_4O_2S_4$ [M+H]+ : 409.0856 Da; found [M+H]+ : 409.0853Da. IR (ATR) $\upsilon^{\sim}$ = 1728 cm$^{-1}$ (C=O), 1119 cm$^{-1}$ (symmetric stretch, C=S) and 1070 cm$^{-1}$ (asymmetric stretch, C=S)

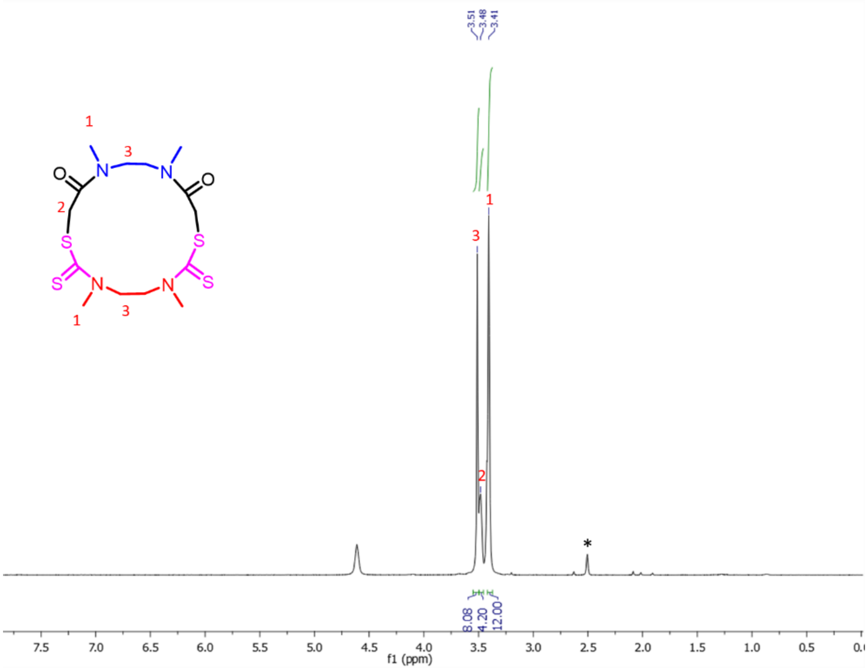


**Figure S16:** $^1$H-NMR (400 MHz) of macrocycle **2** in DMSO-d$_6$

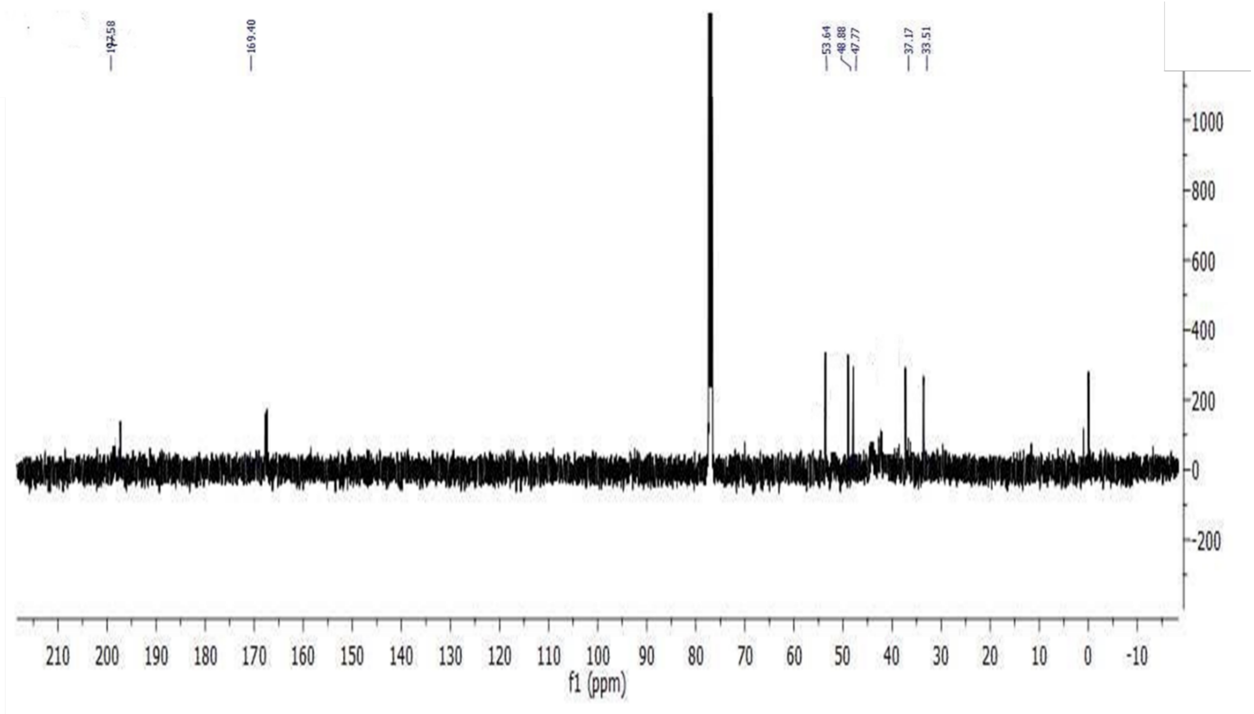


**Figure S17:** $^{13}$C-NMR of macrocycle **2** in $CDCl_3$

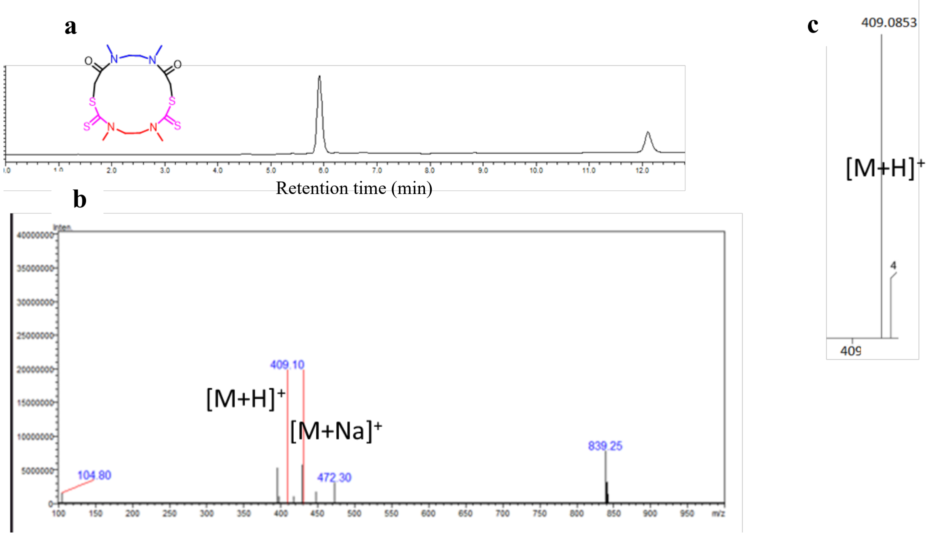


**Figure S18 a:** HPLC of macrocycle **2** eluted in acetonitrile and water gradient recorded at 254 nm. **b**: Full range MS spectrum in positive ion mode. **c**: HRMS with $[M+H]^+$ peak

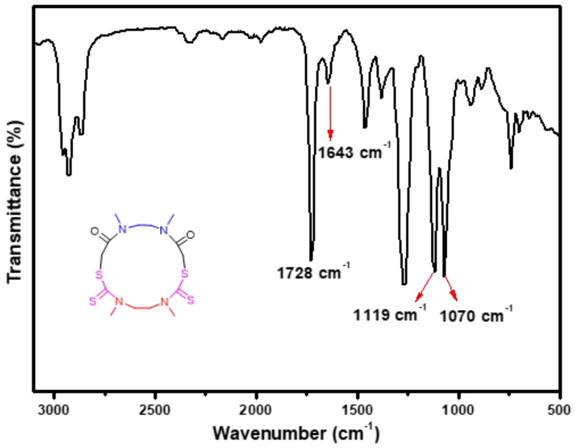


**Figure S19:** FT-IR spectrum of macrocycle **2.**

*4.3.3 Macrocycle **3***

The macrocycle is synthesized by following the procedure given and purified by column chromatography, isolated as white solid (78.5%). $^{1}$H NMR (500 MHz, $CDCl_3$ δ (ppm) 2.13 (m, J= 20 Hz, 2H), 3.14 (s, 6H), 3.62 (m, J= 20 Hz, 4H), 3.67 (s, 4H), 3.99 (s, 4H), 7.73 (s). "#" and "*" represent the residual proton of internal standard tetramethyl silane and $CDCl_3$ respectively. $^{13}$C NMR (125 MHz, $CDCl_3$) δ 29.87, 36.42, 45.23, 46.65, 61.34, 166.12, 185.13. LC-MS calculated $[M+H]^+$: 395.06 Da, observed $[M+H]^+$: 395.60 Da, $[M+Na]^+$: 417.90 Da. HRMS (ESI) m/z calculated for $C_{14}H_{24}N_4O_2S_4$ $[M+H]^+$ : 395.0700 Da; found [M+H]+ : 395.0648 Da. IR (ATR) ῦ = 1643 cm$^{-1}$, 1728 cm$^{-1}$ (C=O), 1101 cm$^{-1}$ (symmetric stretch, C=S) and 1070 cm$^{-1}$ (asymmetric stretch, C=S)

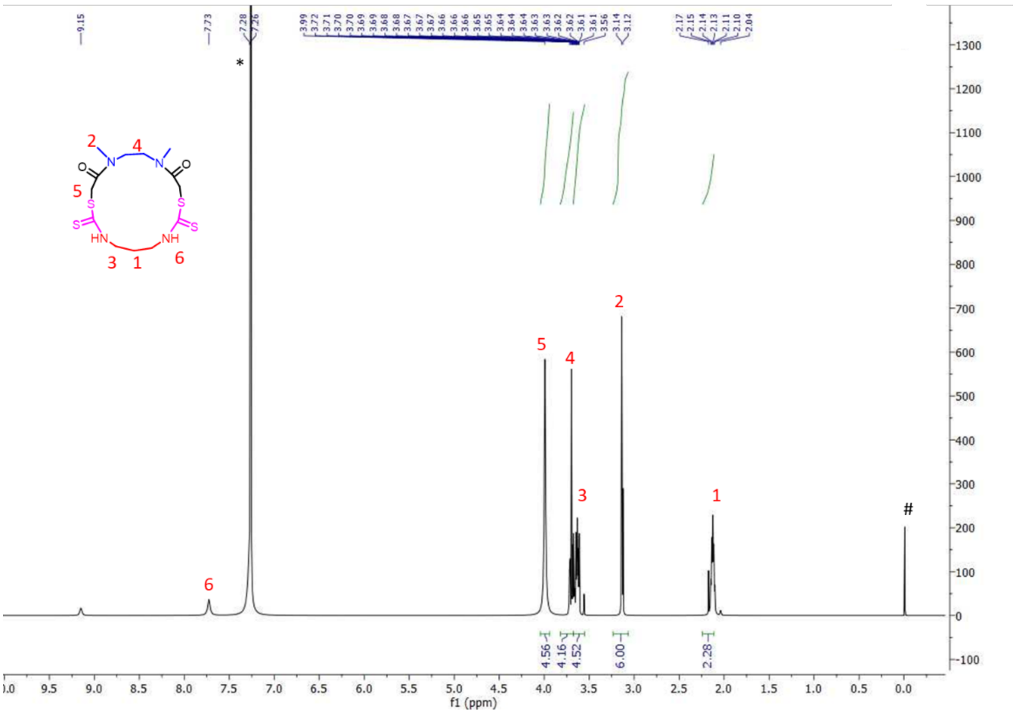


**Figure S20:** $^{1}$H-NMR (500 MHz) of macrocycle **3** in $CDCl_3$

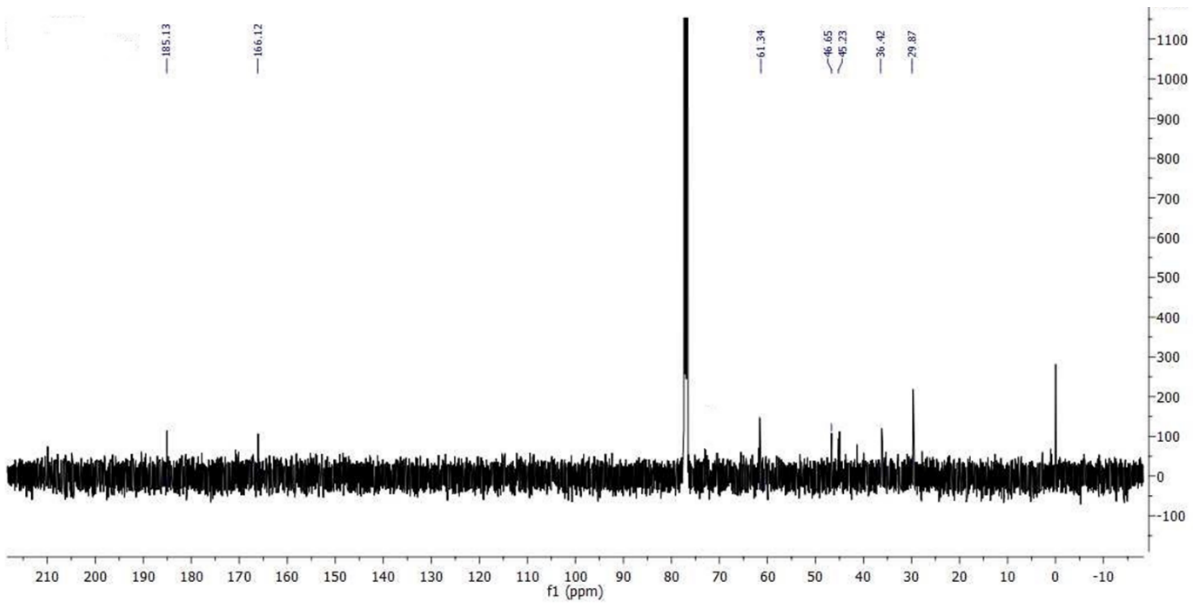


**Figure S21:** $^{13}$C-NMR of macrocycle **3** in $CDCl_3$

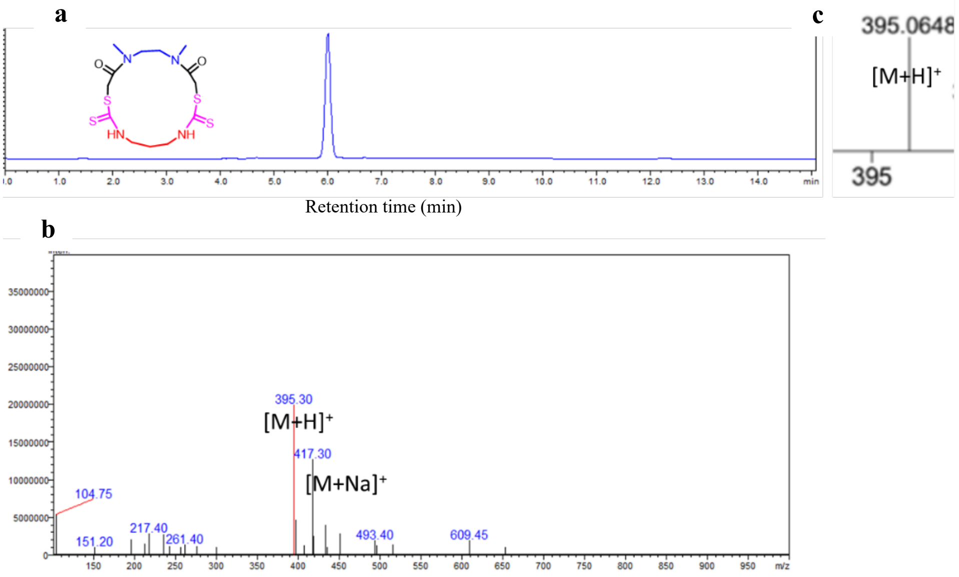


**Figure S22 a**: HPLC of macrocycle **3** eluted in acetonitrile and water gradient recorded at 254 nm. **b**: Full range MS spectrum in positive ion mode. **c**: HRMS with $[M+H]^+$ peak

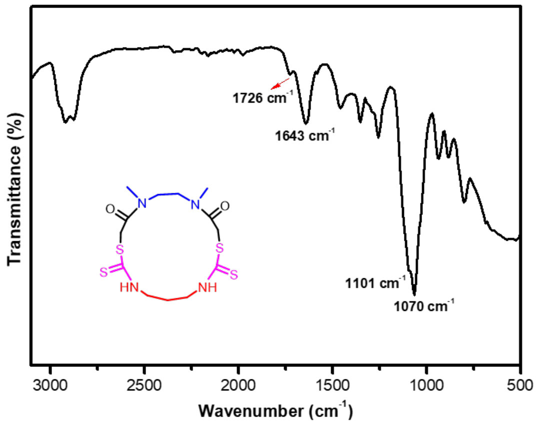


**Figure S23:** FT-IR spectrum of macrocycle **3.**

*4.3.4 Macrocycle* ***4***

The macrocycle is synthesized by following the procedure given and purified by column chromatography, isolated as yellow oil (85.3%). $^1$H NMR (400 MHz, $CDCl_3$) δ (ppm) 3.63 (t, J = 4.0 Hz, 4H), 3.68 (s, 4H), 3.72 (t, J= 8 Hz, 8H), 8.18(s). "#" and "*" represent the residual proton signal of internal standard tetramethyl silane and $CDCl_3$ respectively.$^{13}$C NMR (125 MHz, $CDCl_3$) δ 23.25, 23.99, 30.64,31.39, 38.76, 168.66, 196.33. LC-MS calculated $[M+H]^+$: 381.25 Da, observed $[M+H]^+$: 381.30 Da, $[M+Na]^+$: 403.65 Da HRMS (ESI) m/z calculated for $C_{12}H_{20}N_4O_2S_4$ [M+H]+ : 381.1543 Da; found [M+H]+ : 381.1519 Da. IR (ATR) υ~ = 1651 cm$^{-1}$ (C=O), 1099 cm$^{-1}$ (symmetric stretch, C=S) and 1061 cm$^{-1}$ (asymmetric stretch, C=S)

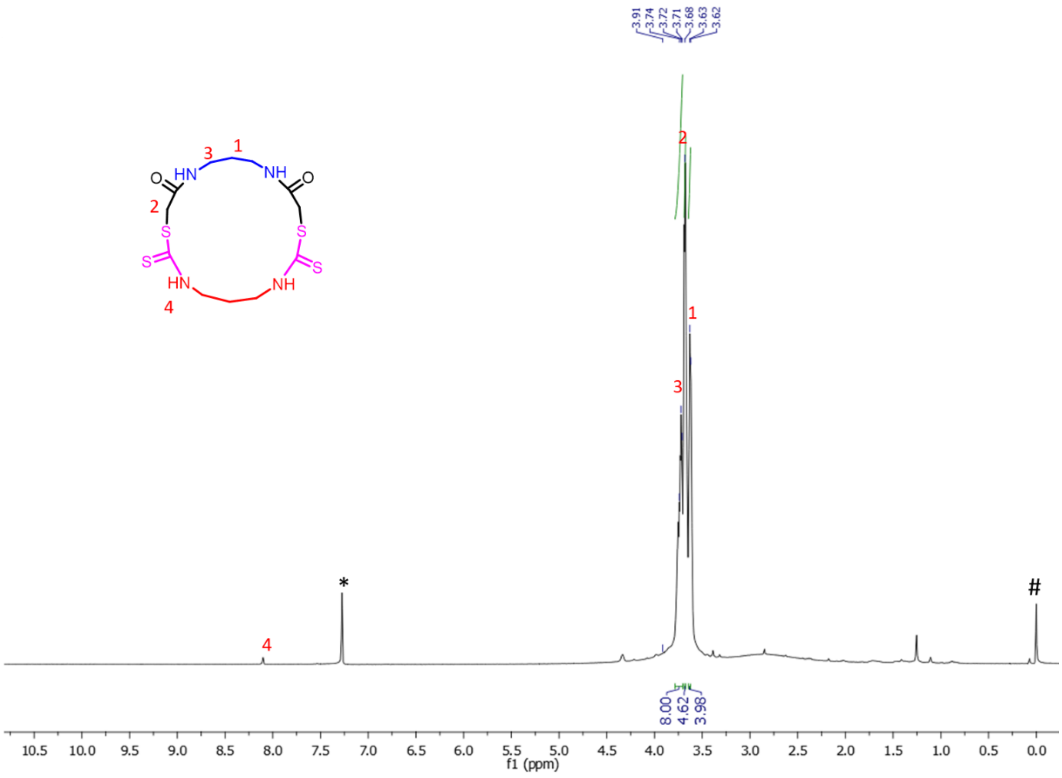


**Figure S24:** $^1$H-NMR (400 MHz) of macrocycle **4** in $CDCl_3$

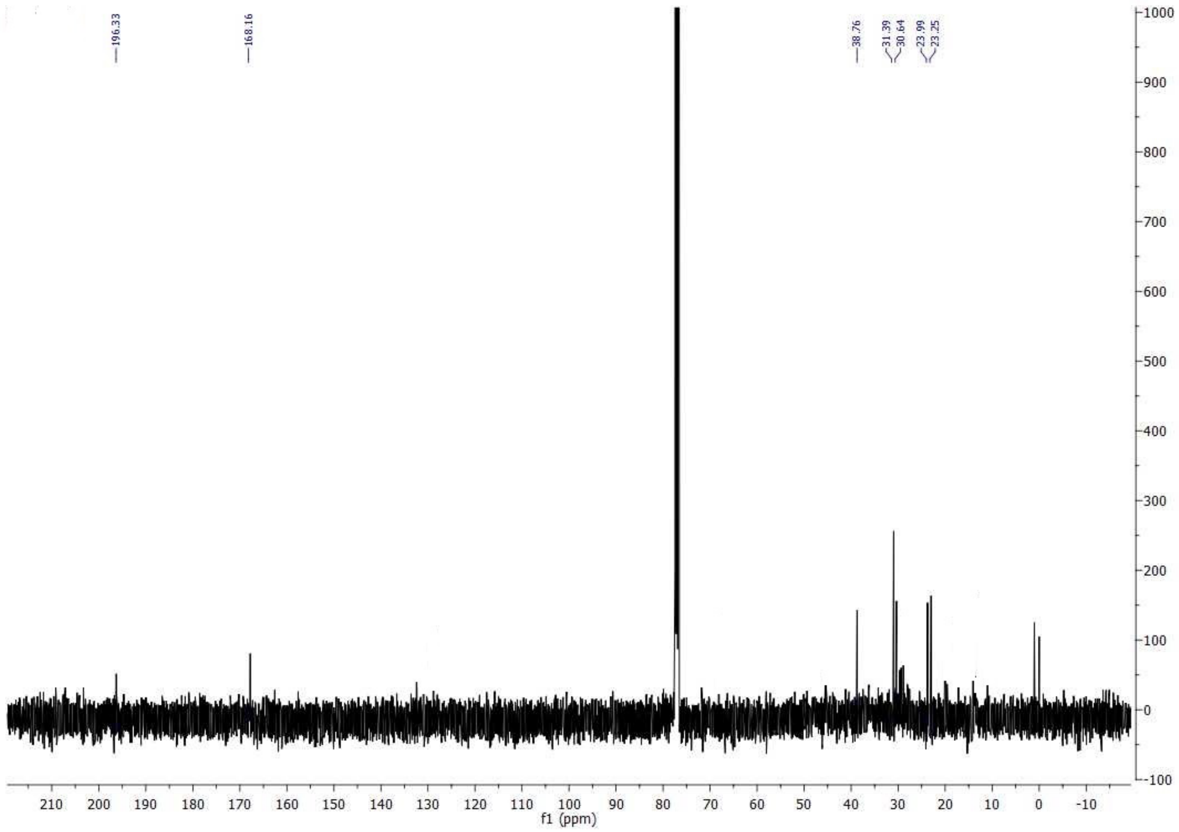


**Figure S25:** $^{13}C$-NMR of macrocycle **4** in $CDCl_3$

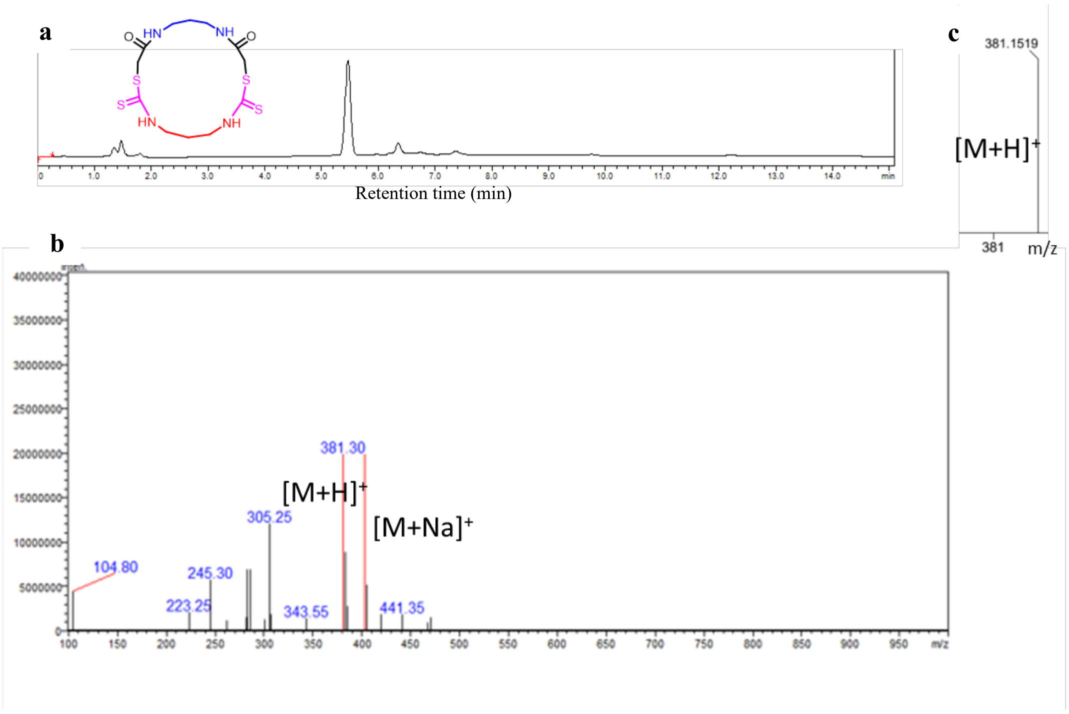


**Figure S26 a:** HPLC of macrocycle **4** eluted in acetonitrile and water gradient recorded at 254 nm. **b**: Full range MS spectrum in positive ion mode. **c**: HRMS with $[M+H]^+$ peak

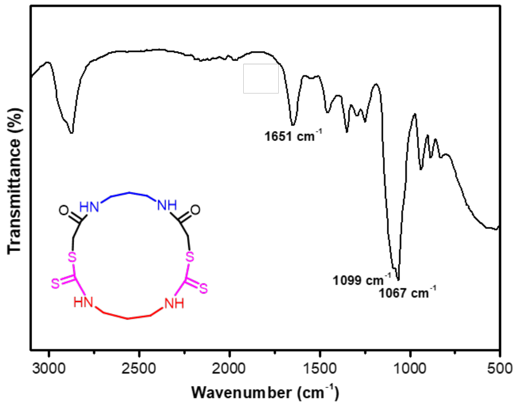


**Figure S27: .** FT-IR spectrum of macrocycle **4.**

*4.3.5 Macrocycle* ***5***

The macrocycle is synthesized by following the procedure given and purified by column chromatography, isolated as yellow oil (74.7%). $^{1}$H NMR (500 MHz, $CDCl_3$) δ (ppm) 1.38 (t, J = 20 Hz, 4H), 2.02 (m, J= 20 Hz, 4H), 2.32 (t, 2H), 3.62 (t, 4H) 3.67 (s, 4H), 3.78 (t, 4H). "#" and "*" represent the residual proton signal of internal standard tetramethyl silane and $CDCl_3$ respectively.$^{13}$C NMR (125 MHz, $CDCl_3$) δ 22.08, 26.20, 27.25, 43.72, 45.97, 54.02, 165.38, 196.84. LC-MS calculated $[M+H]^+$: 423.09 Da, observed $[M+H]^+$: 423.40 Da, $[M+Na]^+$: 445.60 Da. HRMS (ESI) m/z calculated for $C_{15}H_{26}N_4O_2S_4$ [M+H]+ : 423.0939 Da; found [M+H]+ : 423.0984 Da. IR (ATR) $\upsilon^{\sim}$ = 1657 $cm^{-1}$, 1729 $cm^{-1}$ (C=O), 1100 $cm^{-1}$ (symmetric stretch, C=S) and 1067 $cm^{-1}$ (asymmetric stretch, C=S)

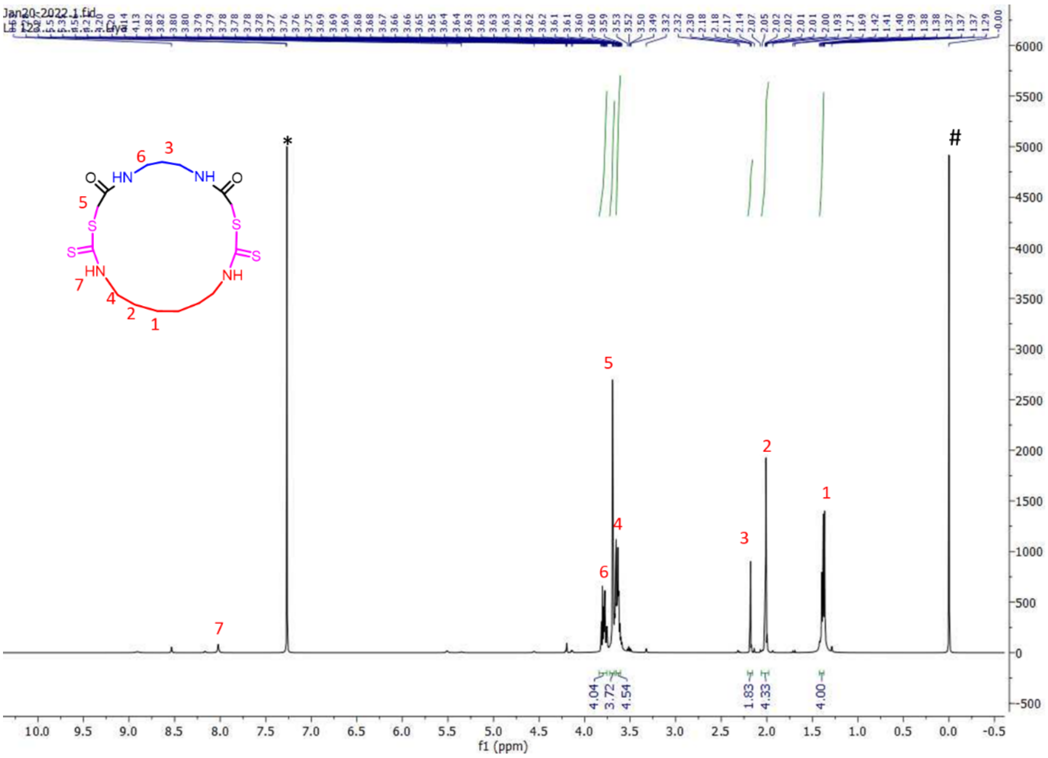


**Figure S28:** $^{1}$H-NMR (500 MHz) of macrocycle **5** in $CDCl_3$

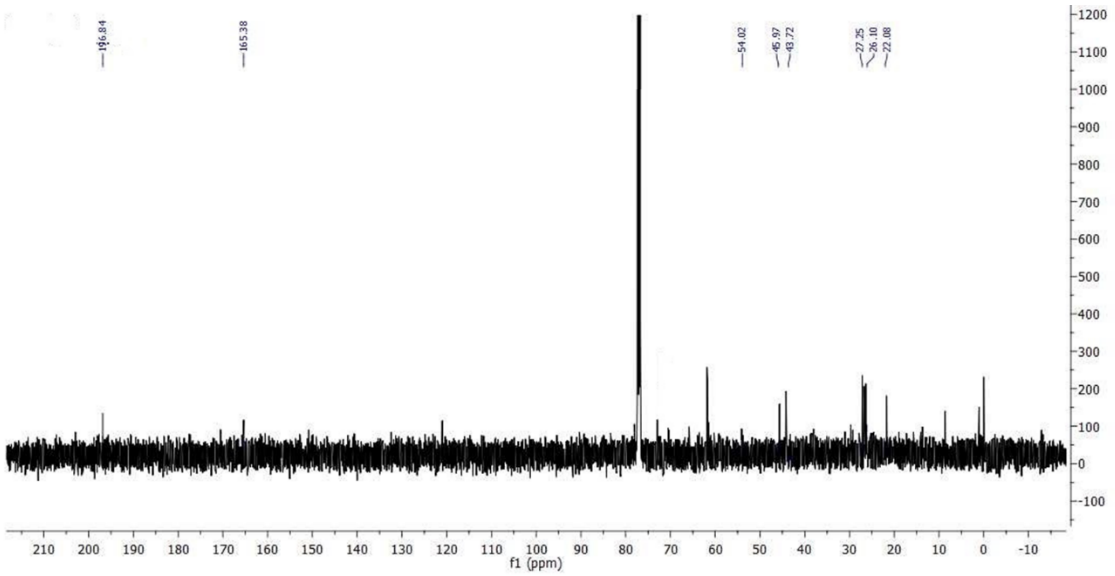


**Figure S29:** $^{13}$C-NMR of macrocycle **5** in $CDCl_3$

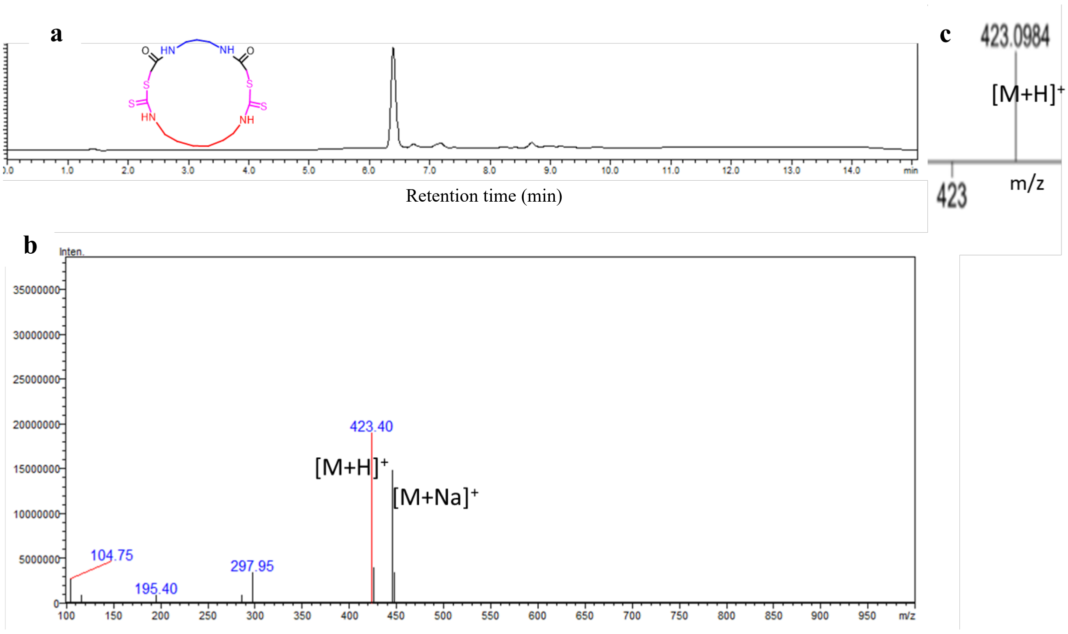


**Figure S30 a:** HPLC of macrocycle **5** eluted in acetonitrile and water gradient recorded at 254 nm. **b**: Full range MS spectrum in positive ion mode. **c**: HRMS with $[M+H]^+$ peak

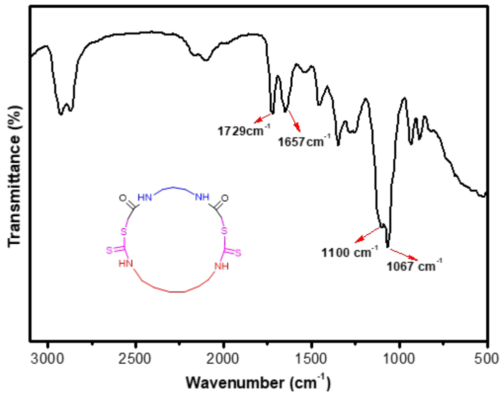


**Figure S31: .** FT-IR spectrum of macrocycle **5.**

*4.3.6 Macrocycle **6***

The macrocycle is synthesized by following the procedure given and purified by column chromatography, isolated as pale-yellow solid (69.4%). $^{1}H$ NMR (500 MHz, $CDCl_3$) δ (ppm) 3.34 (t, J= 20 Hz, 2H), 3.63 (s, 4H), 4.07 (t, J= 10 Hz, 4H), 4.68 (s, 4H), 7.16-7.42 (m, 10H), 8.60 (s), "#" and "*" represent the residual proton signal of internal standard tetramethyl silane and $CDCl_3$ respectively. $^{13}C$ NMR (125 MHz, $CDCl_3$) δ 31.38, 52.07, 56.16, 63.88, 125.71, 126.76, 127.53, 128.27, 131.96, 168.54, 194.84. LC-MS calculated $[M+H]^+$: 547.12 Da, observed $[M+H]^+$: 547.21 Da, $[M+Na]^+$: 569.46 Da. HRMS(ESI) m/z calculated for $C_{25}H_{30}N_4O_2S_4$ [M+H]+ : 547.1326 Da; found [M+H]+ : 547.1332 Da. IR (ATR) $\tilde{\upsilon}$ = 1651 $cm^{-1}$, 1722 $cm^{-1}$ (C=O), 1126 $cm^{-1}$ (symmetric stretch, C=S) and 1070 $cm^{-1}$ (asymmetric stretch, C=S)

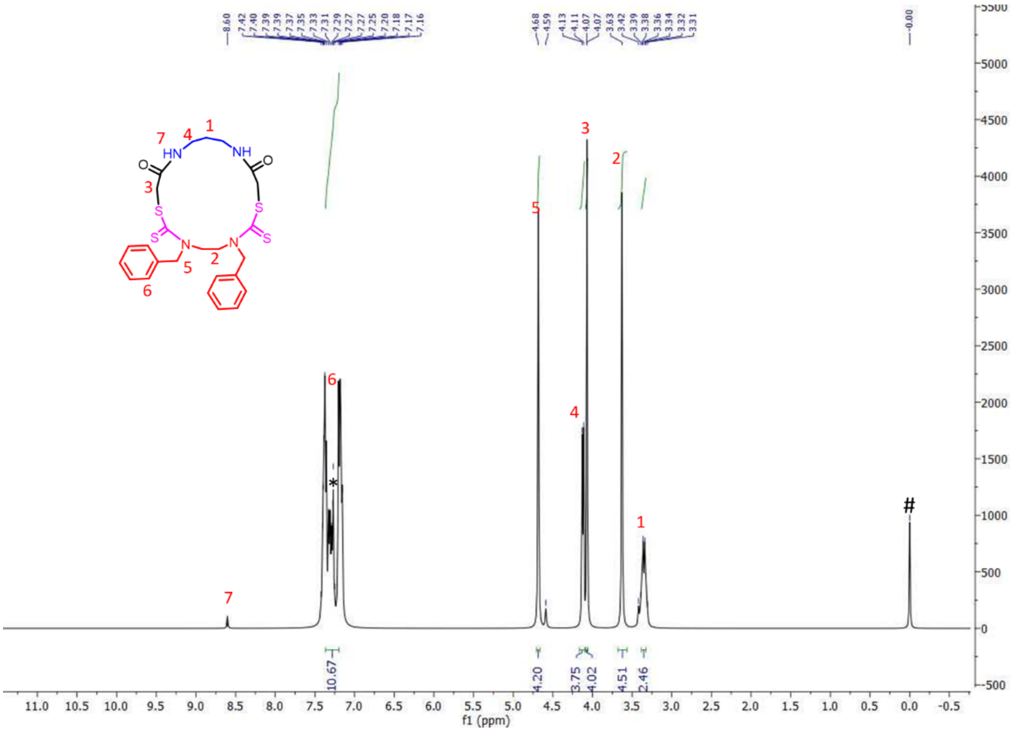


**Figure S32:** $^{1}H$-NMR (500 MHz) of macrocycle **6** in $CDCl_3$

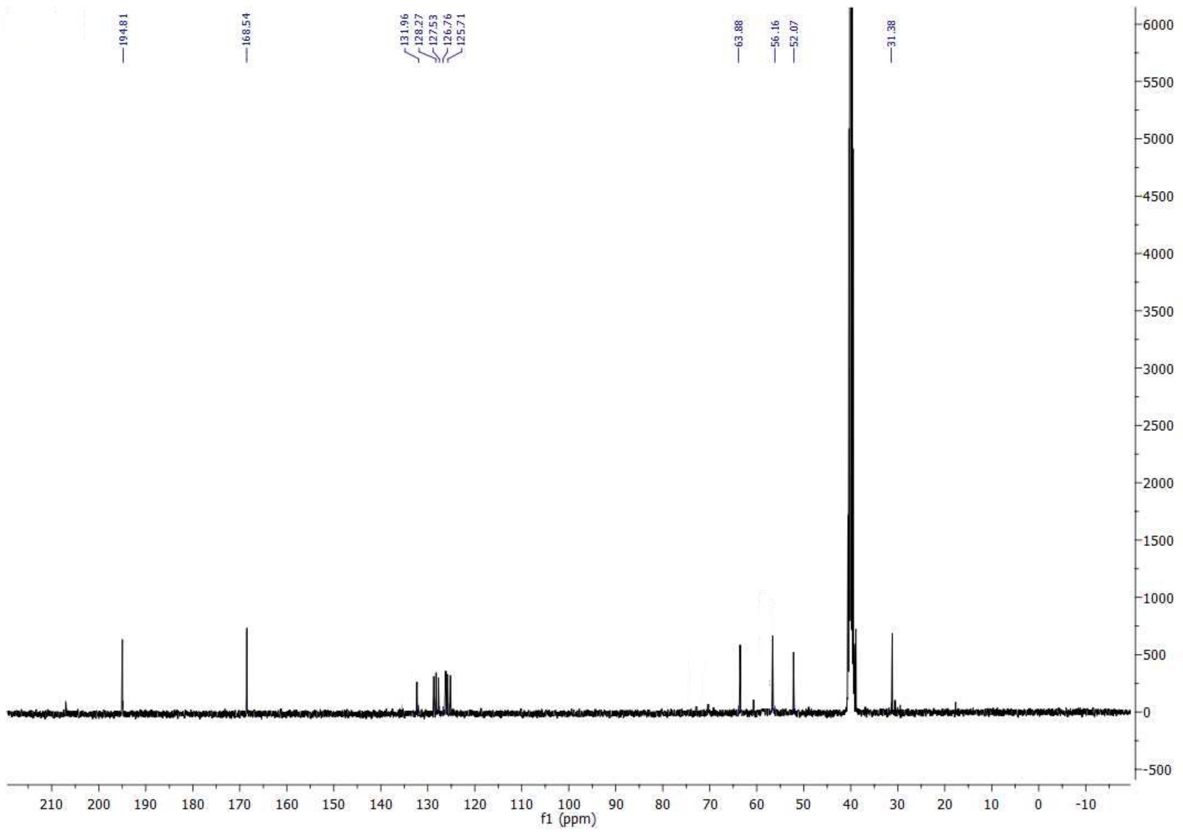


**Figure S33:** $^{13}$C-NMR of macrocycle **6** in DMSO-$d_6$

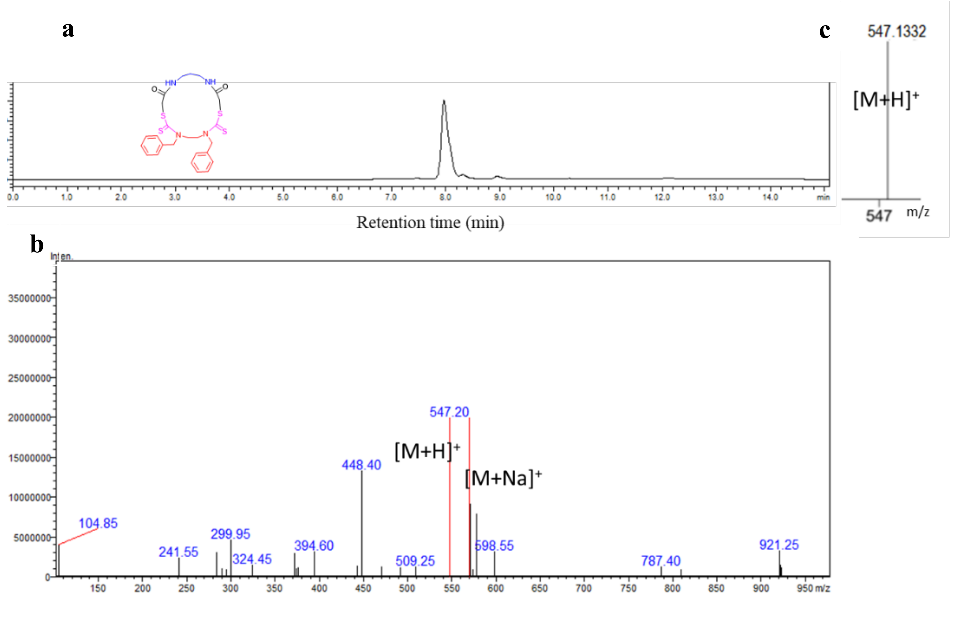


**Figure S34 a:** HPLC of macrocycle **6** eluted in acetonitrile and water gradient recorded at 254 nm. **b**: Full range MS spectrum in positive ion mode. **c**: HRMS with $[M+H]^+$ peak

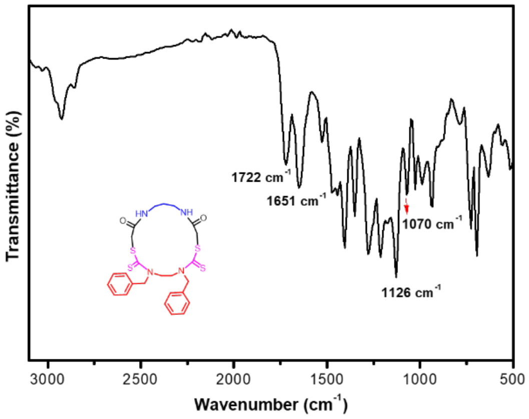


**Figure S35: .** FT-IR spectrum of macrocycle **6.**

*4.3.7 Macrocycle 7*

The macrocycle is synthesized by following the procedure given and purified by column chromatography, isolated as white solid (84.6%). $^1$H NMR (500 MHz, $CDCl_3$) δ (ppm) 3.01 (s, 6H), 3.15 (t, J= 8 Hz, 2H), 3.48 (s, 4H), 3.66 (t, J= 10 Hz), 4.04 (s, 4H), 7.35-7.98 (m, 7H), 8.10 (s), "#" and "*" represent the residual proton signal of internal standard tetramethyl silane and $CDCl_3$ respectively.$^{13}$C NMR (125 MHz, $CDCl_3$) δ 38.95, 40.09, 47.38, 48.49, 51.80, 53.64, 121.37, 125.47, 127.26, 128.00, 129.49, 129.79, 168.28, 169.41, 197.58. LC-MS calculated $[M+H]^+$: 507.09 Da, observed $[M+H]^+$: 507.25 Da. $[M+Na]^+$: 529.50 Da. HRMS(ESI) m/z calculated for $C_{22}H_{26}N_4O_2S_4$ [M+H]+ : 507.1013 Da; found [M+H]+ : 507.1400. IR (ATR) υ˜ = 1643 $cm^{-1}$ (C=O), 1118 $cm^{-1}$ (symmetric stretch, C=S) and 1079 $cm^{-1}$ (asymmetric stretch, C=S)

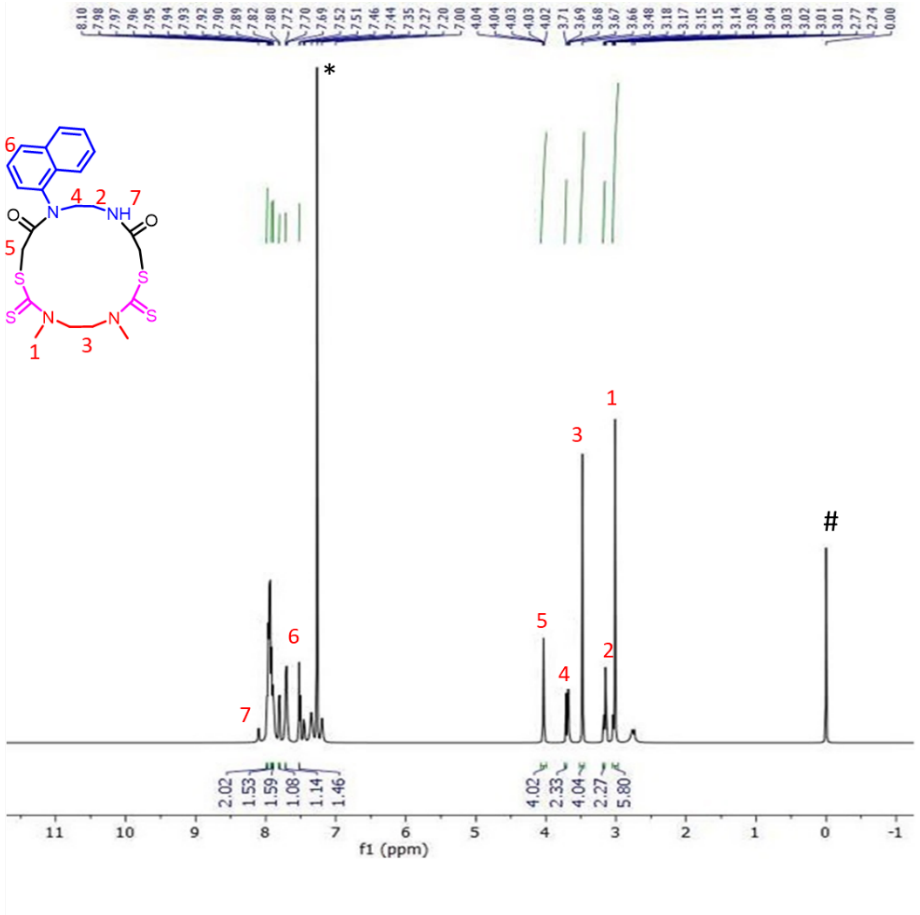

**Figure S36:** $^{1}$H-NMR (500 MHz) of macrocycle **7** in $CDCl_3$

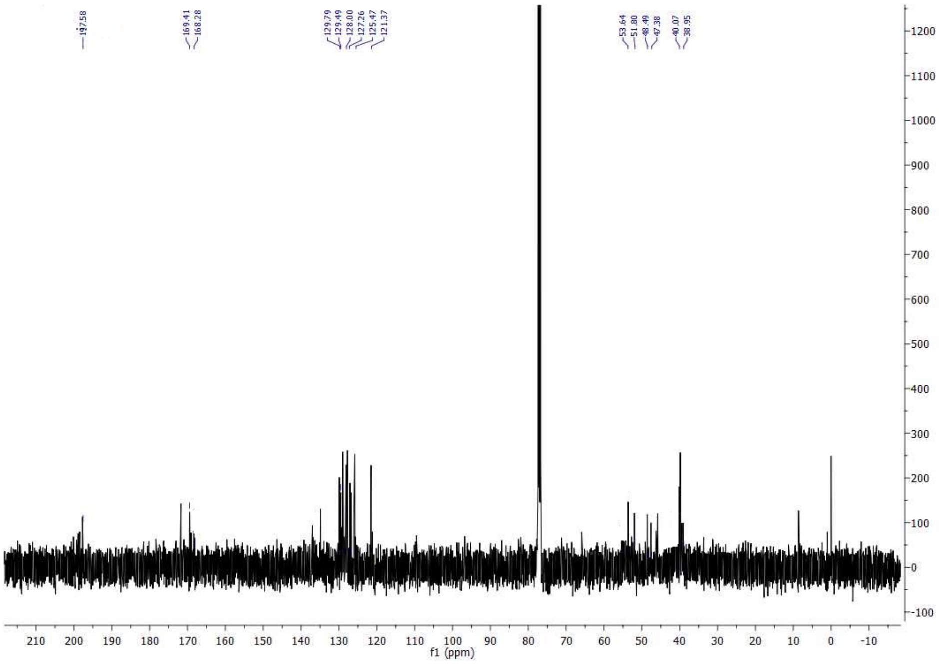

**Figure S37:** $^{13}$C-NMR of macrocycle **7** in $CDCl_3$

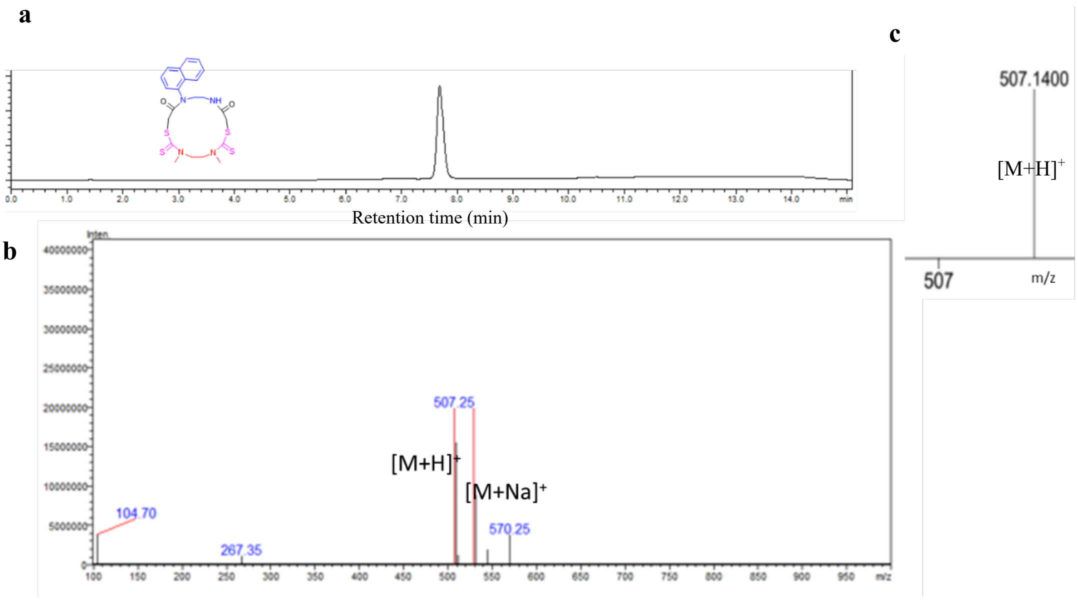


**Figure S38 a:** HPLC of macrocycle **4** eluted in acetonitrile and water gradient recorded at 254 nm. **b**: Full range MS spectrum in positive ion mode. **c**: HRMS with $[M+H]^+$ peak

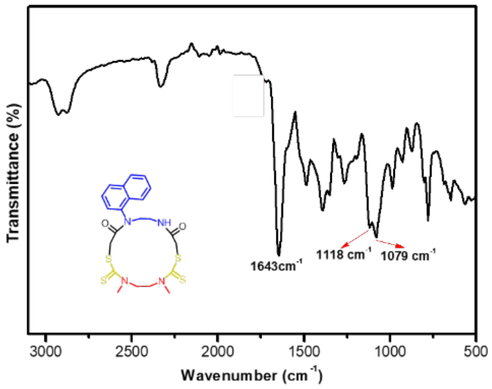


**Figure S39: .** FT-IR spectrum of macrocycle **7.**

5. Scalability

The gram scale synthesis of macrocycle **4** was carried and 1.85 g of pure product was obtained. The image of the product is shown below.

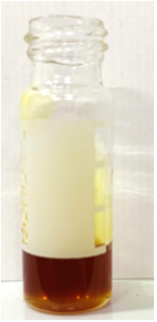

**Figure S40.** 1.85g of macrocycle

6. Druggability and protein interaction studies

6.1 Physicochemical properties

The druggability of the synthesized macrocycles are tested by calculating the physicochemical properties by using the webtool SWISSADME[4] the properties are compared with the conventional guidelines for drug and the guidelines for macrocyclic drug proposed by Whitty and coworkers[5]

**Table S2.** Physicochemical properties of the macrocycles: MW – Molecular weight, clogP – lipophilicity, PSA – Polar surface area, HBD – Hydrogen bond donors, HBA – Hydrogen bond acceptors, RB – Rotatable bonds.

| Property | Conventional drug[6] | Macrocyclic drug[5] | **1** | **2** | **3** | **4** | **5** | **6** | **7** |
|---|---|---|---|---|---|---|---|---|---|
| MW | ≤500 | 600-1200 | 492.77 | 408.61 | 394.59 | 380.57 | 422.64 | 546.78 | 506.73 |
| clogP | ≤5 | -2 to 6 | 2.98 | 0.99 | 0.80 | 0.72 | 1.60 | 3.21 | 2.84 |
| PSA (A°) | ≤140 | 180-320 | 161.88 | 161.88 | 179.46 | 197.04 | 197.04 | 179.46 | 170.67 |
| No. of HBD | ≤5 | ≤12 | 0 | 0 | 2 | 4 | 4 | 2 | 1 |
| No. of HBA | ≤10 | 12-16 | 2 | 2 | 2 | 2 | 2 | 2 | 2 |
| No. of RB | ≤10 | ≤15 | 6 | 0 | 0 | 0 | 0 | 4 | 1 |

6.2 Determination of binding energy and docked conformation from molecular docking

**Table S3.** Binding energy of the synthesized macrocycles and a positive control on interaction with BSA and HSA

| Macrocycle | Binding energy (kcal/mol) |
|---|---|
| **1** | -5.8 |
| **2** | -5.3 |
| **3** | -6.5 |

| | |
|---|---|
| **4** | -6.3 |
| **5** | -6.4 |
| **6** | -7.4 |
| **7** | -7.1 |
| Ibuprofen | -7.3 |

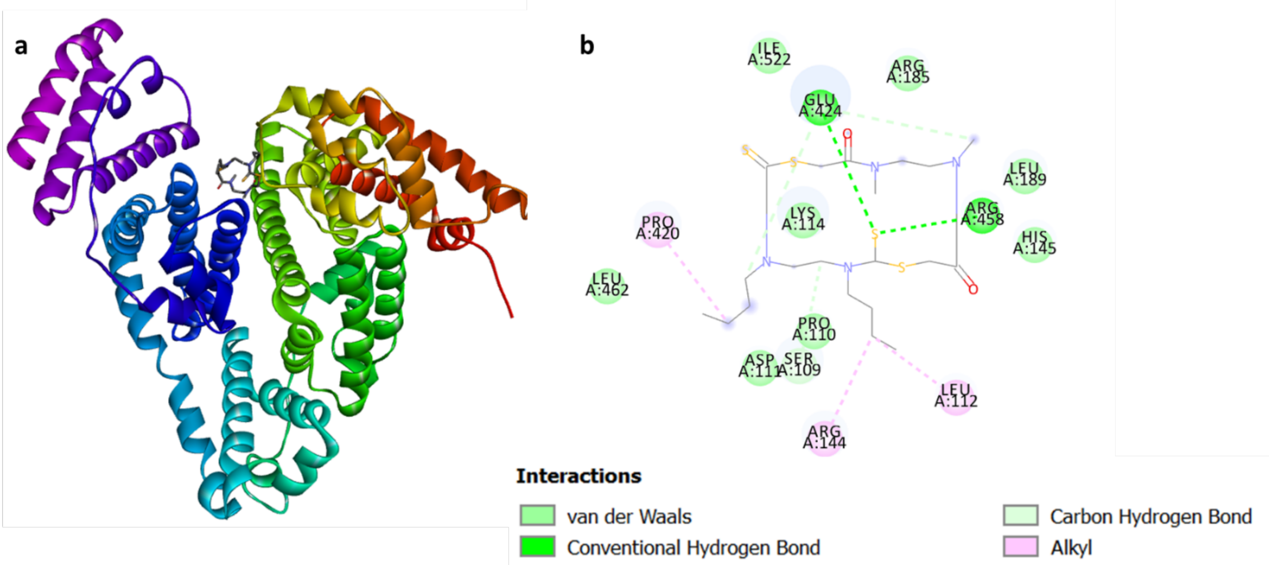


**Figure S41 a.** The best docked conformation of the macrocycle **1** with the BSA. **b.** 2D diagram of aminoacid interaction of BSA complexed with macrocycle **1**.

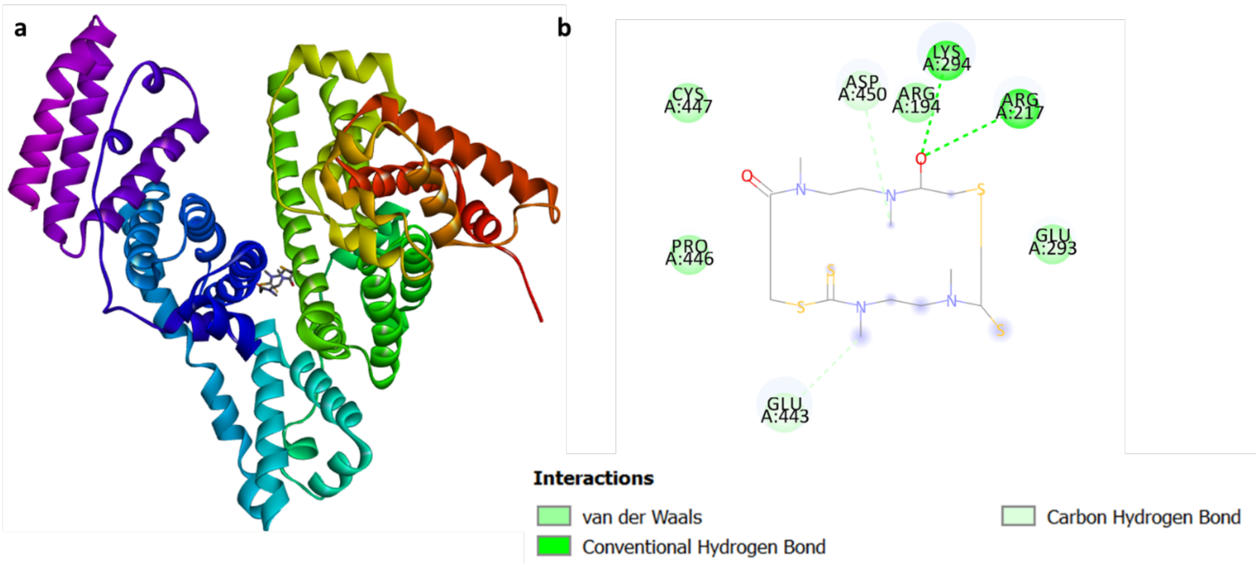


**Figure S42 a.** The best docked conformation of the macrocycle **2** with the BSA. **b.** 2D diagram of aminoacid interaction of BSA complexed with macrocycle **2**.

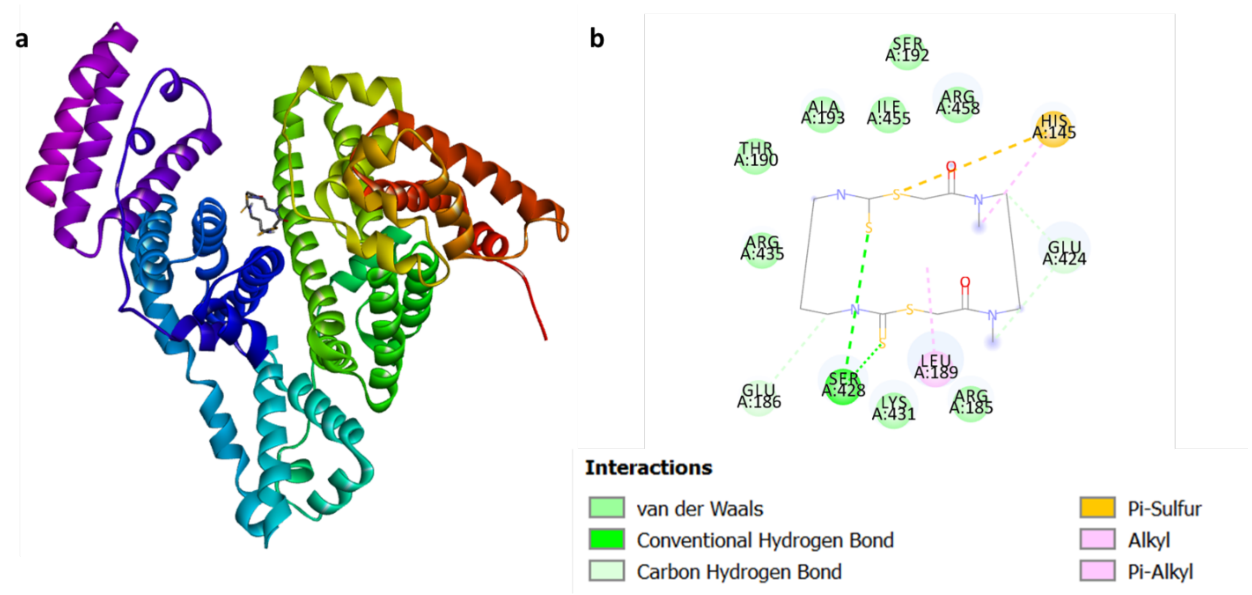


**Figure S43 a.** The best docked conformation of the macrocycle **3** with the BSA. **b.** 2D diagram of aminoacid interaction of BSA complexed with macrocycle **3**.

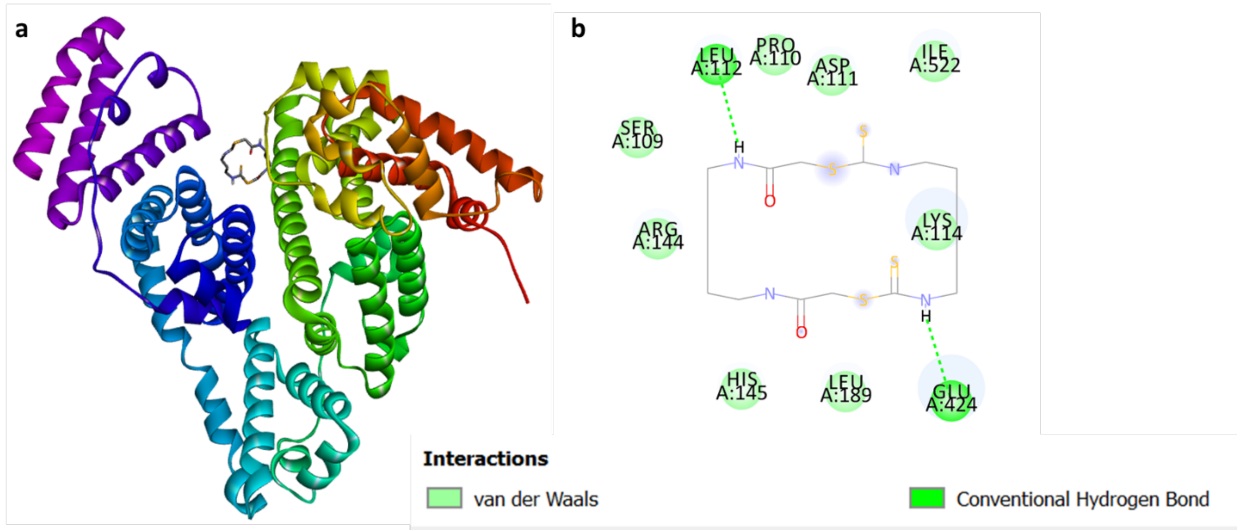


**Figure S44 a.** The best docked conformation of the macrocycle **4** with the BSA. **b.** 2D diagram of aminoacid interaction of BSA complexed with macrocycle **4**.

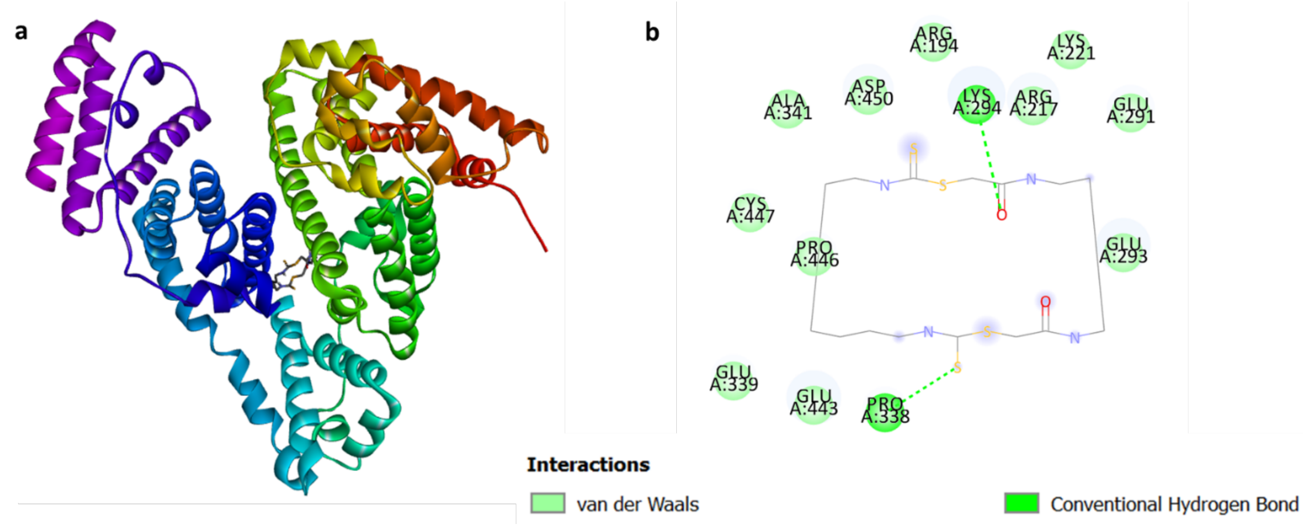


**Figure S45 a.** The best docked conformation of the macrocycle **5** with the BSA. **b.** 2D diagram of aminoacid interaction of BSA complexed with macrocycle **5**.

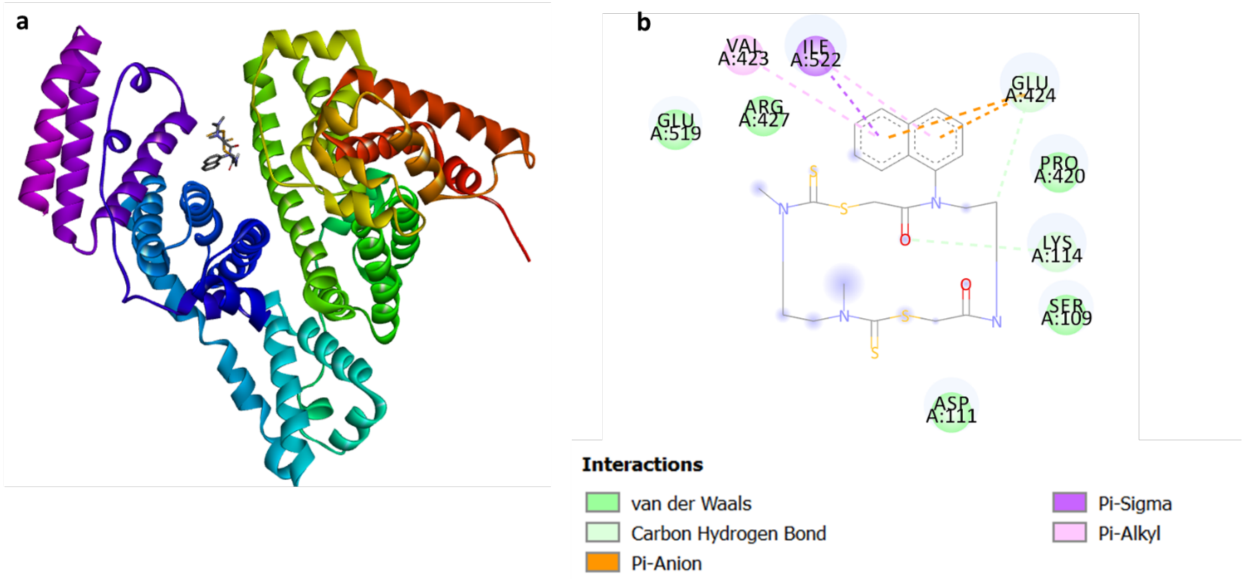


**Figure S46 a.** The best docked conformation of the macrocycle **7** with the BSA. **b.** 2D diagram of aminoacid interaction of BSA complexed with macrocycle **7**.

6.3 Fluorescence spectroscopy for protein interaction studies

*6.3.1 Experimental procedure*

10 µM BSA was prepared by dissolving 6.6mg of BSA in 10 ml of phosphate buffer of pH 7.4. The macrocycles were dissolved in DMSO to make the stock solution of 2 mM concentration. 2 ml of the BSA control solution was taken in the fluorescence cuvette to take the emission at zero concentration of the compound. The titration was carried out by varying the concentration of macrocycle from 2 µM to 100 µM. An equilibration time of 3 minutes is given for each measurement after the addition of the solution. Binding constant were estimated from the Stern-

Volmer plot by plotting $I_o/I$ vs concentration of the macrocycle. The slope of the graph was the attributed as the binding constant.

### *6.3.2 Fluorescence spectra for the interaction of BSA and macrocycles*

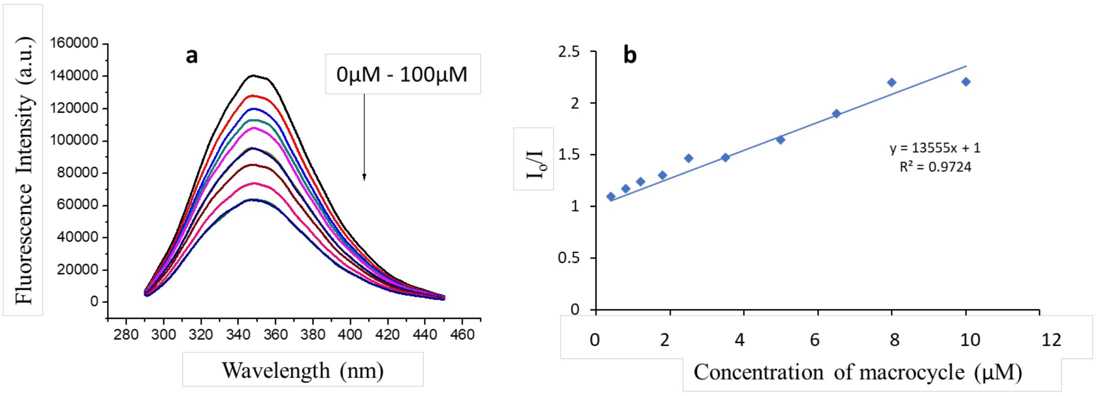


**Figure S47. a.** Fluorescence spectrum of macrocycle **1** on interaction with BSA. **b.** The plot of $I_o/I$ vs concentration of macrocycle. The slope of the graph is attributed as binding constant, which is (13.55 ± 0.72) x$10^3$ $M^{-1}$.

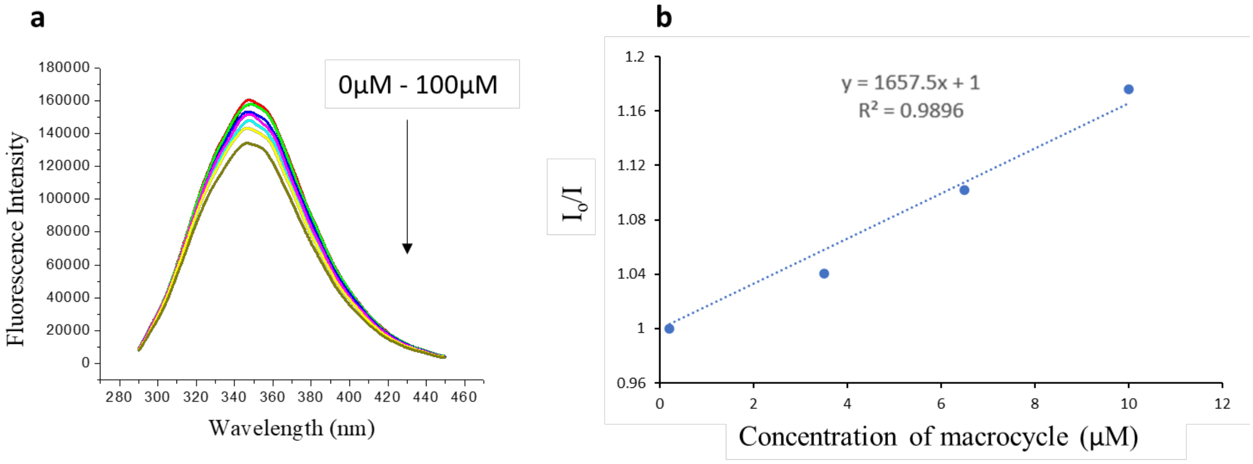


**Figure S48. a.** Fluorescence spectrum of macrocycle **2** on interaction with BSA. **b.** The plot of $I_o/I$ vs concentration of macrocycle. The slope of the graph is attributed as binding constant, which is (1.66 ± 0.32) x$10^3$ $M^{-1}$.

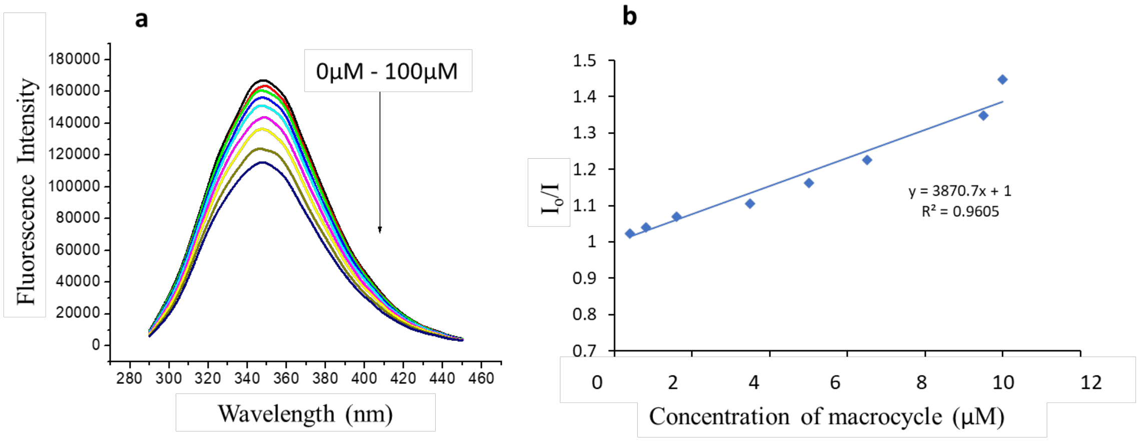


**Figure S49. a.** Fluorescence spectrum of macrocycle **3** on interaction with BSA. **b.** The plot of $I_o/I$ vs concentration of macrocycle. The slope of the graph is attributed as binding constant, which is $(3.87 \pm 0.33)$ x$10^3$ $M^{-1}$.

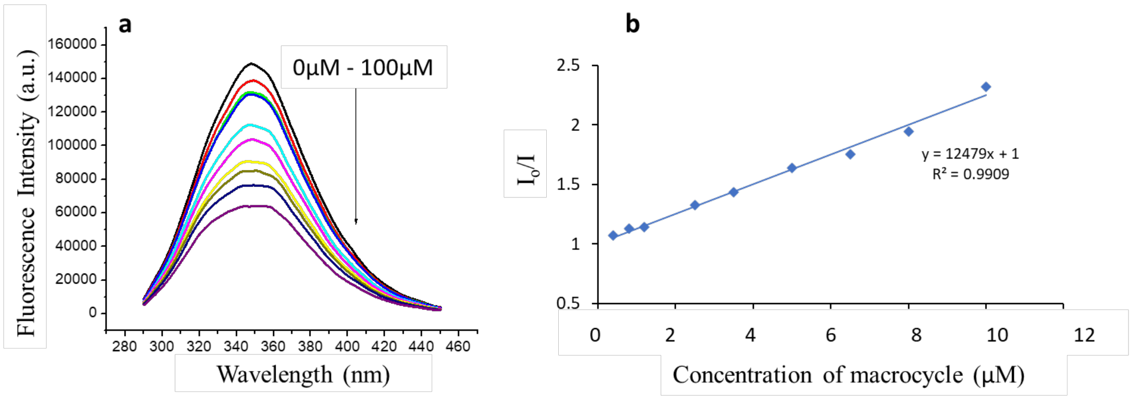


**Figure S50. A.** Fluorescence spectrum of macrocycle **4** on interaction with BSA. **B.** The plot of $I_o/I$ vs concentration of macrocycle. The slope of the graph is attributed as binding constant, which is $(12.48 \pm 0.45)$ x$10^3$ $M^{-1}$.

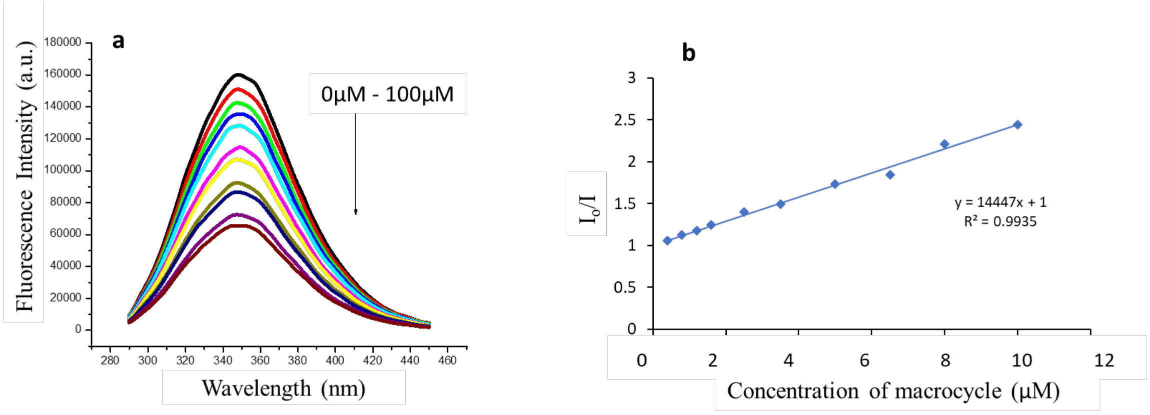


**Figure S51. a.** Fluorescence spectrum of macrocycle **5** on interaction with BSA. **b.** The plot of $I_o/I$ vs concentration of macrocycle. The slope of the graph is attributed as binding constant, which is $(14.45 \pm 0.68)$ x$10^3$ $M^{-1}$.

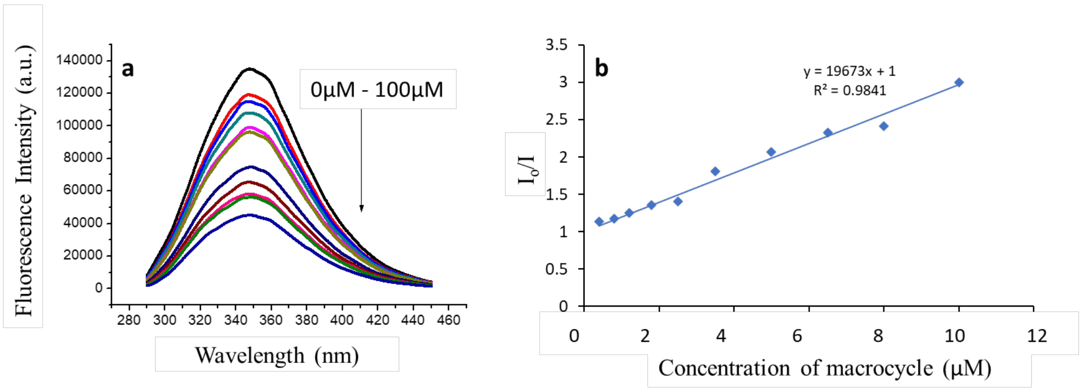


**Figure S52. a.** Fluorescence spectrum of macrocycle **7** on interaction with BSA. **b.** The plot of $I_o/I$ vs concentration of macrocycle. The slope of the graph is attributed as binding constant, which is $(19.67 \pm 0.86)$ x$10^3$ $M^{-1}$.

## 7. Supporting References

1. Morris, G.M. *et al*. AutoDock4 and AutoDockTools4: Automated docking with selective receptor flexibility, *J. Comput. Chem*. **30,** 2785–2791 (2009)
2. Trott, O., Olson, A., AutoDock Vina: improving the speed and accuracy of docking with a new scoring function, efficient optimization, and multithreading, *J. Comput. Chem.* **31** 455–461, (2010)
3. Ranjith, D. Molecular docking studies of aloe vera for their potential antibacterial activity using Argus lab 4.0. 1." *The Pharma Innovation Journal* **8**, 481-487, (2019)
4. Daina, A., Michielin, O., & Zoete, V. SwissADME: a free web tool to evaluate pharmacokinetics, drug-likeness and medicinal chemistry friendliness of small molecules. *Sci Rep.*, **7**, 1-13, (2017).
5. Villar, E. A. et al., How proteins bind macrocycles. *Nat. Chem. Biol.* **10,** 723-731, (2014).
6. Lipinski, C. A., Lombardo, F., Dominy, B. W. & Feeney, P. J. Experimental and computational approaches to estimate solubility and permeability in drug discovery and development settings. *Adv. Drug Deliv. Rev.* **23**, 3–25 (1997).
